# Highest Solar-to-Hydrogen Conversion Efficiency in Cu_2_ZnSnS_4_ Photocathodes and Its Directly Unbiased Solar Seawater Splitting

**DOI:** 10.1007/s40820-025-01755-8

**Published:** 2025-05-16

**Authors:** Muhammad Abbas, Shuo Chen, Zhidong Li, Muhammad Ishaq, Zhuanghao Zheng, Juguang Hu, Zhenghua Su, Yanbo Li, Liming Ding, Guangxing Liang

**Affiliations:** 1 Institute of Thin Film Physics and Applications, Shenzhen Key Laboratory of Advanced Thin Films and Applications, Key Laboratory of Optoelectronic Devices and Systems of Ministry of Education and Guangdong Province, State Key Laboratory of Radio Frequency Heterogeneous Integration, College of Physics and Optoelectronic Engineering, Shenzhen University, Shenzhen, 518060 People’s Republic of China; 2 Institute of Fundamental and Frontier Sciences, University of Electronic Science and Technology of China, Chengdu, 610054 People’s Republic of China; 3 School of Chemical Engineering and Light Industry, Guangdong University of Technology, Guangzhou, 510006 People’s Republic of China

**Keywords:** Cu_2_ZnSnS_4_, Solar-to-hydrogen conversion, Charge transfer dynamics, Precursor seed layer engineering, Seawater splitting

## Abstract

Despite being an excellent candidate for a photocathode, Cu_2_ZnSnS_4_ (CZTS) performance is limited by suboptimal bulk and interfacial charge carrier dynamics. In this work, we introduce a facile and versatile CZTS precursor seed layer engineering technique, which significantly enhances crystal growth and mitigates detrimental defects in the post-sulfurized CZTS light-absorbing films. This effective optimization of defects and charge carrier dynamics results in a highly efficient CZTS/CdS/TiO_2_/Pt thin-film photocathode, achieving a record half-cell solar-to-hydrogen (HC-STH) conversion efficiency of 9.91%. Additionally, the photocathode exhibits a highest photocurrent density (*J*_ph_) of 29.44 mA cm^−2^ (at 0 *V*_RHE_) and favorable onset potential (*V*_on_) of 0.73 *V*_RHE_. Furthermore, our CTZS photocathode demonstrates a remarkable *J*_ph_ of 16.54 mA cm^−2^ and HC-STH efficiency of 2.56% in natural seawater, followed by an impressive unbiased STH efficiency of 2.20% in a CZTS-BiVO_4_ tandem cell. The scalability of this approach is underscored by the successful fabrication of a 4 × 4 cm^2^ module, highlighting its significant potential for practical, unbiased in situ solar seawater splitting applications.

**Supplementary Information:**

The online version contains supplementary material available at 10.1007/s40820-025-01755-8.

## Highlights

- A novel approach, precursor seed layer engineering, is applied to prepare Cu_2_ZnSnS_4_ (CZTS) light-absorbing films using the solution-processed spin-coating method.
- Mo/CZTS/CdS/TiO_2_/Pt photocathode effectively mitigates defects in CZTS light absorber and the CZTS/CdS heterojunction interface, optimizing charge carrier dynamics.
- Record photoelectrochemical performance including half-cell solar-to-hydrogen (HC-STH) efficiency of 9.91%, photocurrent density of 29.44 mA cm^−2^ at 0 *V*_RHE_ in 0.5 M H_2_SO_4_ electrolyte, and STH efficiency of 2.20% in CZTS-BiVO_4_ tandem cell in natural seawater is achieved.

**Supplementary Information:**

The online version contains supplementary material available at 10.1007/s40820-025-01755-8.

## Graphical abstract

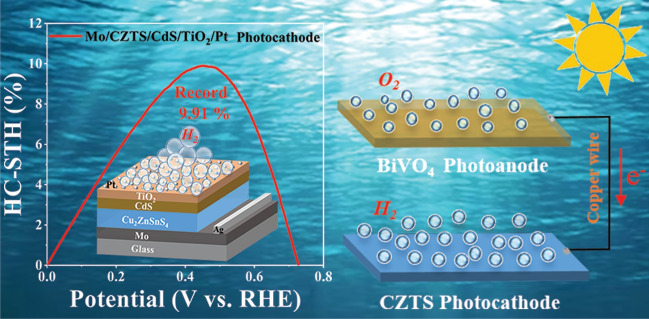

## Introduction

In the modern world, fossil fuels, which are non-renewable and bad for the environment, are still the most widely used energy source that is required to be replaced to improve the standard of living on our planet. Therefore, future energy shortages are expected to be eased by hydrogen energy, a new environmentally friendly green energy source. Most of the current methods for producing hydrogen use a significant amount of electricity and cause CO_2_ emissions, highlighting the need for cleaner approaches such as solar-to-hydrogen (STH) conversion via photoelectrochemical (PEC) water splitting [1]. It has been widely believed that PEC water splitting using sunlight irradiation is a clean way to produce hydrogen energy. Building upon the pioneering work of Honda and Fujishima [2], researchers have explored a wide range of semiconductor materials as a photoelectrode, to enhance their STH conversion efficiency, photocurrent density (*J*_ph_), onset potential (*V*_on_), and stability [3, 4]. The semiconductor absorber layer and absorber/buffer layer’s heterojunction form the core of PEC water splitting devices (photocathodes), where crucial processes such as charge production, transfer, separation, and recombination occur [5]. For example, the effectiveness of production of charge carriers in the p-type absorber layer and n-type buffer layer, as well as the subsequent transfers and collection of these carriers at the heterojunction interface, directly influences the current density and overall efficiency of the photocathode.

P-type semiconductors, especially chalcogenides, such as Cu_3_BiS_3_ [6], CuGaSe_2_ [7], Cu_2_ZnSn(S,S)_4_ (CZTSSe) [5], Cu_2_BaSn(S,Se)_4_ [8], CuSbS_2_ [9], Sb_2_Se_3_ [10], CdTe [11], and Cu(In,Ga)Se_2_ (CIGS) [12], commonly utilized in photovoltaic applications, are preferred as photocathodes due to their appropriate optical band gaps and high light absorption coefficients. Despite facing challenges in large-scale production and low STH conversion efficiency and current density, there has been significant improvements in Cu-chalcogenide compound semiconductors in recent years. Notably, a 9.30% half-cell solar-to-hydrogen (HC-STH) conversion efficiency has already been achieved by CIGS-based thin-film photocathodes [13], accompanied by high *J*_ph_ of 35.5 mA cm^−2^ [14], but their utilization of scarce elements like indium and gallium poses limitations for widespread domestic applications. Furthermore, CZTSSe emerged as promising semiconductor material for photocathode [8]. Despite improvements in *J*_ph_, the STH efficiency and *V*_on_ of CZTSSe photocathodes remain relatively low due to challenges associated with low charge carrier generation and high recombination rates caused by antisite/vacancy defects within the CZTSSe absorber layer and interface defects at CZTSSe/CdS heterojunction [5]. Therefore, by effectively managing the recombination and generation of charge carriers, as well as addressing bulk and heterojunction defects, it is anticipated that the PEC properties of chalcogenide compound-based photocathodes can be significantly enhanced.

Hence, researchers, including our team, are actively investigating novel low-cost and environmentally friendly materials, with a focus on chalcogenide compounds due to their demonstrated high potential in maintaining superior PEC properties [5, 15]. We believe that CZTS-based photocathodes exhibit immense potential, leveraging their inherent exceptional natural properties, including absorption coefficient (> 10^4^ cm^−1^), accompanied by a band gap range of 1.45–1.56 eV [16, 17], highlighting their potential for exceptional sunlight absorption. Meanwhile, its earth-abundant elements and low toxicity make it conducive to practical application [18]. Since the pioneering work of Domen’s group in 2010, which underscored the significance of CZTS photocathodes in water splitting [19], CZTS has made significant strides in overcoming various challenges related to efficiency improvement and long-term durability [4, 20–24]. Subsequently, the highest *J*_ph_, *V*_on_, and HC-STH efficiency of CZTS-based photocathodes are reported to be 29 mA cm^−2^ at 0 *V*_RHE_, 0.75 *V*_RHE_ [25], and 7.27% [26], respectively, demonstrating promising performance in this field. Despite the considerable research efforts, substantial hurdles still persist in effectively addressing the issues to further enhance their PEC properties. Issues such as suboptimal charge carrier dynamics, interfacial recombination, and susceptibility to corrosion in harsh environments continue to limit their performance and durability.

While diverse factors influence the PEC performance of CZTS photocathodes, carrier generation and recombination dynamics near the CZTS/CdS heterojunction, coupled with defect-mediated recombination in the junction region and bulk CZTS, are central to better device performance [27]. Notably, impurities such as deep-level and shallow defects within the CZTS absorber’s band gap, along with interfacial defects at the CZTS/CdS boundary, are also the key contributors to recombination losses [28]. Prior studies have explored these challenges through post-sulfurization treatments [29–33], identifying Cu_Zn_ antisite defects and copper vacancies (*V*_Cu_) with shallow acceptor levels as dominant recombination centers. Despite these insights, progress has remained incremental. It is important to recognize that these issues are closely tied to the structural and morphological quality of CZTS thin films, such as crystallinity, grain uniformity, interfacial integrity with the substrate (back contact), and buffer layer compatibility (front contact). Therefore, to advance PEC performance, it is critical to optimize charge generation, separation, and collection processes, enhance carrier transport pathways, and reduce defect-related losses. Achieving this requires careful consideration of CZTS preparation steps, from precursor seed layer synthesis to sulfurization, alongside a deeper understanding of defect behavior and carrier dynamics.

In this work, we develop a facile and versatile precursor seed layer engineering (PSLE) approach during the solution-processed preparation of the CZTS precursor seed layer. We demonstrate that optimizing the precursor layer prior to sulfurization is a key strategy for addressing persistent challenges associated with CZTS thin films. Through meticulous optimization of the hot-plate annealing durations, we observed a thermodynamic nucleation evolution in the precursor films, presenting film morphology changed from clustered-like structures to well-separated granular structures. By focusing on these transformations, which critically govern the kinetic recrystallization and growth mechanisms of CZTS thin films during sulfurization, we establish a pathway to achieve high-quality CZTS thin-film light absorbers. The resulting CZTS absorber layers exhibit well-aligned and smooth grain boundaries, without the bulk and cross-sectional pinholes or cracks. Ultimately, the impact of PSLE strategy on the CZTS absorber layer and CZTS/CdS heterojunction serves to suppress defect densities and defects-assisted non-radiative recombination, extend carrier lifetime, intensify the generation and separation of photo-generated charge carriers, and ultimately enhance the PEC performance of the CZTS-based thin-film photocathodes. A record-breaking 9.91% HC-STH efficiency of PSLE-dependent planar-type Mo/CZTS/CdS/TiO_2_/Pt photocathode was achieved, accompanied with simultaneously high *J*_ph_ of 29.44 mA cm^−2^ (at 0 *V*_RHE_) and *V*_on_ of 0.73 *V*_RHE_. Furthermore, one of the highest *J*_ph_ of 16.54 mA cm^−2^ (at 0 *V*_RHE_), *V*_on_ of 0.78 *V*_RHE_, and a considerable HC-STH efficiency of 2.56% provide a way to bright future of natural solar seawater splitting. Finally, we fabricated a CZTS-BiVO_4_ tandem cell for unbiased solar seawater splitting, achieving an unassisted STH conversion efficiency of 2.20%. To demonstrate its scalability, a 4 × 4 cm^2^ large-area tandem cell module was successfully developed, highlighting its significant potential for practical and scalable overall solar seawater splitting applications.

## Experimental Section

### Materials

All chemicals were used without further refining. The cuprous chloride (CuCl, ≥ 99.95%), zinc acetate dihydrate (C_4_H_6_O_4_Zn·2H_2_O, 99.99%), stannic chloride hydrated (SnCl_4_·5H_2_O, 99.99%), and 2-methoxyethanol (C_3_H_8_O_2_, ≥ 99.7%) were purchased from Aladdin Reagents, and tin(II) sulfide (SnS, 99%), sublimated sulfur (S, 99.95%), ammonia solution (NH_3_), cadmium sulfate (CdSO_4_, 99%), thiourea (CH_4_N_2_S, ≥ 99%), and titanium(IV) chloride (TiCl_4_, ≥ 99%) were purchased from Sigma-Aldrich.

### Synthesis of CZTS Light Absorber Thin Film

CZTS thin films were fabricated using a solution-processed spin-coating method. The precursor solution was prepared by dissolving CuCl (1.52 g), C_4_H_6_O_4_Zn 2H_2_O (2.1951 g), SnCl_4_·5H_2_O (2.9217 g), CH_4_N_2_S (4.8717 g), and C_3_H_8_O_2_ (10 mL) under continuous stirring until all constituents were fully dissolved. Subsequently, an additional 10 mL of C_3_H_8_O_2_ and 5 mL of the yellow-colored precursor solution were mixed and carefully filtered. The resulting precursor solution was deposited onto Mo-coated substrate using spin-coating method. The commercially available Mo-coated glass substrates were cleaned in an ultrasonic bath using the following sequence: detergent for 20 min, ethanol for 10 min, and deionized water (DI water) for 20 min. After cleaning, the substrates were vacuum-dried in an oven at 60 °C for 20 min. Each sample was spin-coated initially at 500 r min^−1^ for 10 s, followed by acceleration to 4000 r min^−1^ for 20 s to ensure uniform film deposition. Subsequently, the samples were annealed on a hot plate using the PSLE technique in an open atmosphere. Through systematic thermal treatment optimization spanning 50 to 350 s, 150 s of annealing duration per cycle over 12 spin-coating cycles emerged as the critical duration for high-quality CZTS films. Conversely, excessively long or short treatment durations resulted in CZTS crystals with either sharp or indistinct edges, leading to films with poor grain connectivity. Following the spin-coating process, post-sulfurization was carried out using a thermally processed sulfurization technique in a vacuum tubular furnace. Sublimed sulfur (0.08321 g) and tin(II) sulfide (0.0535 g) were placed in a covered graphite box along with four CZTS precursor-layered samples, which were then positioned inside the furnace tube. The sulfurization process involved a two-step rapid thermal treatment: First, the temperature was raised to 80 °C at a heating rate of 4 °C s⁻^1^, followed by a rapid increase to 620 °C at a heating rate of 5 °C s⁻^1^. The samples were maintained at 620 °C for 20 min before being allowed to cool spontaneously to room temperature.

### Fabrication of Mo/CZTS/CdS/TiO_2_/Pt Thin-Film Photocathodes

Following the preparation of the CZTS light-absorbing thin film, the CdS buffer layer was chemically bath deposited (CBD) using a KW-4A desktop chemical bath model. DI H_2_O (140 mL), NH_3_ (14.8 M, 21 mL), CH_4_N_2_S (0.75 M, 20 mL), and CdSO_4_ (0.015 M, 20 mL) were introduced one after the other to a particular container holding SLG/Mo/CZTS samples. The container was covered and then placed in a water bath at 80 °C with slow stirring for 8 min and 30 s. The SLG/Mo/CZTS/CdS samples were then cleaned with deionized water and dried in an oven at 60 °C. The NaFeng NCR-200R atomic layer deposition (ALD) machine was utilized to deposit the protective layer of TiO_2_ on top of the CdS layer. The TiCl_4_ and H_2_O were used as the titanium and oxygen precursors, accordingly. The growth rate of 0.7 Å each cycle was maintained to control the thickness of TiO_2_ at 10 nm. Finally, a 108 Auto Sputter Coater was used to introduce Pt co-catalyst for 50 s at a DC current of 20 mA. Consequently, a thin-film photocathode with the structure SLG/Mo/CZTS/CdS/TiO_2_/Pt was fabricated. Prior to evaluating the device performance, commercially available silver (Ag) electrodes were manually painted onto the exposed surface of the Mo substrate, following its etching, to establish ohmic contact.

### Fabrication of BiVO_4_ Photoanode and CZTS-BiVO_4_ Tandem Device

BiVO_4_ films were prepared by electrodepositing BiOI precursor onto a cleaned FTO glass substrate using a three-electrode system. It was then converted to BiVO_4_ by applying a vanadium source solution, followed by the electrodeposition of a CoPi co-catalyst to construct BiVO_4_ photoanode, the detailed preparation process can be found in Note S1. Finally, a custom-built CZTS-BiVO_4_ tandem device was assembled by connecting a 4 × 4 cm^2^ CZTS photocathode and a 4 × 4 cm^2^ BiVO_4_ photoanode with copper wires.

### PEC Measurements

The PEC performance was evaluated using a CHI 660E electrochemical workstation and a standard three-electrode model, with a Pt-rod serving as the counter electrode, Ag/AgCl in saturated KCl solution serving as the reference electrode, and the SLG/Mo/CZTS/CdS/TiO_2_/Pt photocathode serving as the working electrode (Fig. S2). The photocathodes were tested in 0.5 M H_2_SO_4_ (pH1) and seawater electrolytes. A solar simulator 150W Xenon Light Source (Gloria-X150A) standardized to 100 mW cm^−2^ with a certified reference cell (Si solar Cell) providing the AM 1.5G simulated sunlight used as a light source. A scan rate of 20 mV s^−1^ was used to measure linear sweep voltammetry (*J-V* curves). Potentials referred to the Ag/AgCl electrode were changed to reversible hydrogen electrodes (*V*_RHE_) by applying the Nernst equation:

1 $$V_{{{\text{RHE}}}} = V_{{\text{Ag/AgCl}}} + 0.059 \times {\text{pH}} + 0.199$$

ZView software was used to fit photoelectrochemical impedance spectroscopy (PEIS) at frequencies ranging from 10 to 10^4^ Hz, which was carried out under simulated sunshine illumination. At a frequency of 1000 Hz, Mott–Schottky (M-S) investigations were performed. The evolved hydrogen and oxygen were assessed using gas chromatography (GC) in a sealed PEC Labsolar 6A work station with GC9790II gas concentration analyzer and PLS-SXE300E Xenon solar simulator. With a 100% Faraday efficiency (FE) assumed, the theoretical quantity of H_2_ and O_2_ produced could be calculated from the photocurrent. The actual amount of H_2_ and O_2_ produced was obtained by measuring the H_2_ and O_2_ concentration. The ratio of real to theoretical H_2_ and O_2_ production was used to determine Faraday efficiency. Meanwhile, the same CHI 660E electrochemical workstation was used to evaluate the PEC performance of CZTS-BiVO_4_ tandem device at unbiased voltage.

### Structural and Defect Characterizations

Using CuK_α_ radiation in an X-ray diffractometer (XRD, Ultima-iv), the structure and crystallinity of CZTS thin films were examined. The surface and cross-sectional morphologies were evaluated using scanning electron microscopy (SEM, Zeiss SUPRA 55), and the topographies and surface potentials of CZTS were investigated using a Kelvin probe force microscope (KPFM, Bruker Dimension ICON). Using FEI Titan Cubed Themis G2 300, transmission electron microscopy (TEM), the internal structure of the CZTS-based photocathode was examined. Further, optical properties of CZTS-1, CZTS-2, and CZTS-3 thin films, including their reflection, transmission, and absorption spectra, were systematically characterized across the wavelength range of 300–1200 nm using a Shimadzu UV-3600 spectrophotometer. Measurements were performed at room temperature under ambient conditions to evaluate their light harvesting potential. Measurements of ultraviolet photoelectron spectroscopy (UPS) at 21.22 eV were made with a PHI 5000 Versa Probe fitted with a He I source. Further analyses, including drive-level capacitance profiling (DLCP), capacitance–voltage (C-V) tests, and device admittance spectroscopy (AS), were performed to elucidate the material properties, charge carrier dynamics, defects information, and performance characteristics. More specifically, C-V tests were performed in the dark, scanning a voltage range of − 0.50 to 0.10 V at a frequency of 10^4^ Hz and with an AC amplitude of 30 mV. A DC voltage of − 0.25 to 0 V was used in conjunction with an AC amplitude ranging from 20 to 140 mV to conduct DLCP analysis. The admittance spectroscopy (AS) curves for the devices were acquired using a Janis VPF-100 closed-cycle cryostat, integrated with a Lake Shore 325 Cryogenic Temperature Controller to regulate temperatures between 300 and 150 K. Measurements were conducted under high-vacuum conditions (< 10^−6^ Torr) to eliminate environmental interference. The frequency-dependent electrical response was systematically probed across a broad frequency range of 10^2^ to 10^6^ Hz using a precision impedance analyzer (e.g., Keysight E4990A), with AC excitation amplitudes kept below 50 mV to ensure linearity. Data were collected at 10 K intervals to resolve thermally activated carrier dynamics, enabling analysis of defect states and interface properties. The setup incorporated shielded coaxial cabling and thermal anchoring to minimize noise and temperature gradients, ensuring reproducible characterization of charge transport mechanisms in the devices.

## Results and Discussion

### Device Performance of PSLE Derived CZTS-Based Photocathodes

**Fig. 1:**
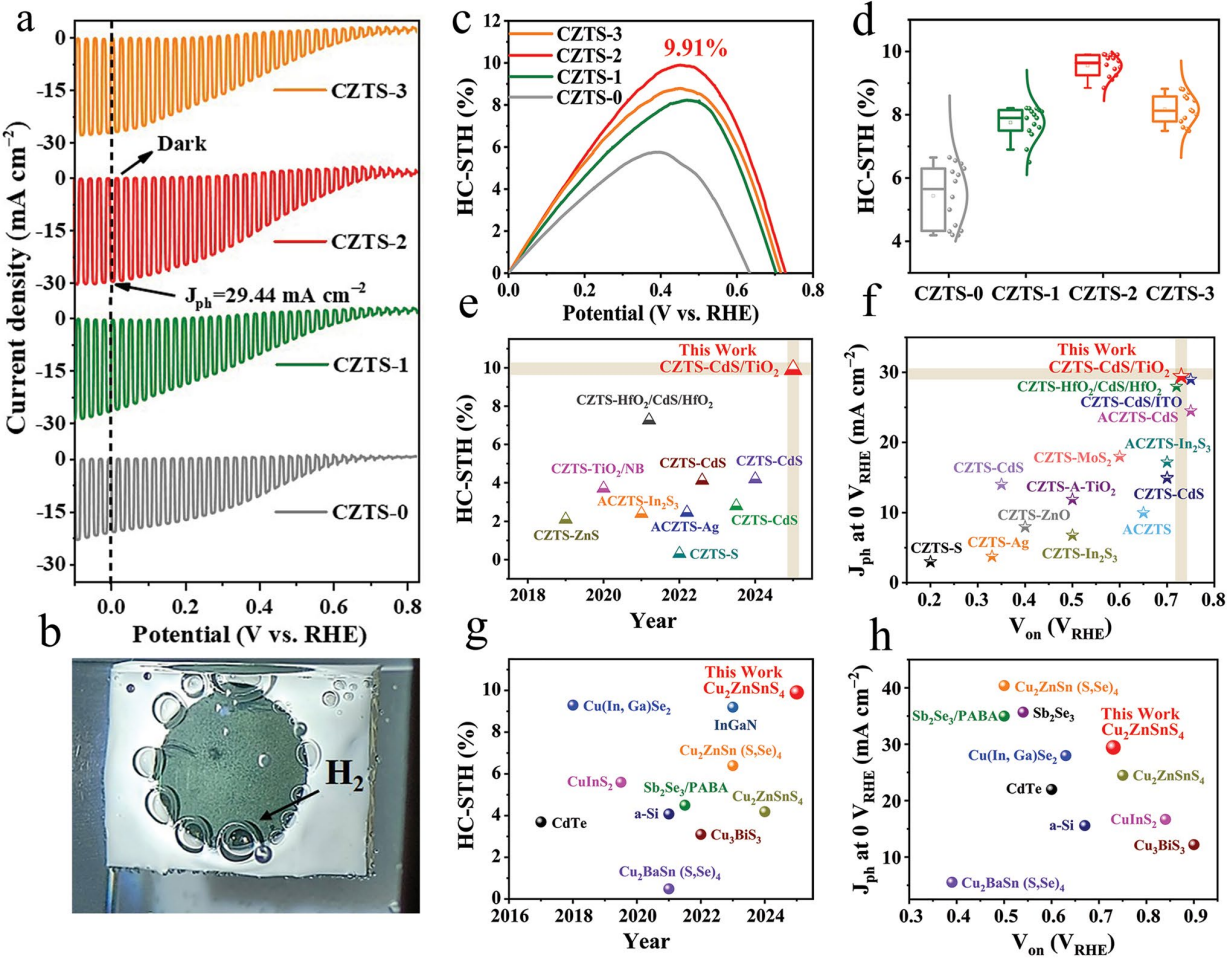
Photoelectrochemical performance of CZTS-based photocathodes. **a** Chopped J-V curves of Mo/CZTS/CdS/TiO_2_/Pt-based, CZTS-0, CZTS-1, CZTS-2, and CZTS-3 photocathodes under dark and AM 1.5G simulated sunlight illumination. **b** A photograph of hydrogen bubbles emerged from photocathode surface to the electrolyte. **c** Calculated HC-STH conversion efficiencies. **d** Statistical distribution and characterizations of HC-STH efficiency of the devices. **e–h** A comparison of this work to those of CZTS-based and state-of-the-art chalcogenide-based photocathodes

CZTS thin films were prepared under hot-plate annealing duration-dependent PSLE process, that is, CZTS-0 of 50 s duration, CZTS-1 of 100 s, CZTS-2 of 150 s, and CZTS-3 of 200 s at fixed temperature of 295 °C (Fig. S1). Corresponding Mo/CZTS/CdS/TiO_2_/Pt photocathodes with an active area of 0.95 cm^2^ were synthesized, and their PEC performance was evaluated using a standard three-electrode PEC water splitting configuration, as presented in our previous works [32, 34], and shown in Fig. S2. Figure 1a illustrates the photocurrent density–potential (J-V) curves evaluated through linear sweep voltammetry (LSV) measurements under chopped sunlight exposure. Specifically, for corresponding CZTS-0, CZTS-1, CZTS-2, and CZTS-3 thin-film photocathodes, the *J*_ph_ values at 0 *V*_RHE_ are determined to be 21.57, 26.50, and 29.44 and 28.19 mA cm^−2^, respectively. Continuous and prominent hydrogen bubbles emerging from the photocathode and electrolyte (i.e., 0.5 M H_2_SO_4_) interface under AM 1.5G simulated sunlight intensity (100 mW cm^−2^) illumination were captured experimentally in a photograph (Fig. 1b), signifying efficient hydrogen evolution reaction (HER) via water splitting. Figure S3 shows the *J-V* curves corresponding to these photocathodes under both continuous simulated sunlight irradiation and dark circumstances. Moreover, the following equation was used to determine the HC-STH conversion efficiency [10]:

2 $$ {\text{HC}} - {\text{STH}}\left( {\text{\% }} \right) = J_{{{\text{ph}}}} \times \left( {V_{{{\text{RHE}}}} - V_{{{\text{H}}^{ + } /{\text{H}}_{2} }} } \right)/P_{{{\text{sun}}}} \times 100{\text{\% }} $$

where ${V}_{\text{RHE}}$ denotes the potential of the photoelectrode, ${J}_{\text{ph}}$ refers to the photocurrent density at the applied potential of ${V}_{\text{RHE}}$, ${V}_{{\text{H}}^{+}/{\text{H}}_{2}}$ signifies the equilibrium redox potential for hydrogen (0 *V*_RHE_), and ${P}_{\text{sun}}$ represents the incident light intensity of 100 mW cm^−2^. The estimated conversion efficiencies of the hydrogen evolution process for each of the corresponding photocathodes are shown in Fig. 1c. In contrast to devices CZTS-0 (0.63 *V*_RHE_), CZTS-1 (0.70 *V*_RHE_), and CZTS-3 (0.71 *V*_RHE_), the HC-STH value of device CZTS-2 is situated at a comparatively higher onset potential of 0.73 *V*_RHE_. This observation suggests a concurrent improvement in photocurrent, onset potential, and overall PEC performance of CZTS-2 photocathode [32]. In parallel, HC-STH efficiency-based statistical distributions CZTS-0, 1, 2, and CZTS-3 photocathodes are shown in Fig. 1d, confirming satisfactory reproducibility in case of CZTS-2 photocathode. The key parameters of our champion photocathode are summed up in Fig. 1e, f and Table S1, which compares it to the previously published CZTS-based photocathodes. Meanwhile, Fig. 1g, h and Table S2 also give a comparison to the previously reported state-of-the-art Si, CdTe, CIGS, and representative chalcogenide photocathodes.

The ground-breaking results achieved in this study, showcasing record HC-STH conversion efficiency of 9.91%, highest *J*_ph_ of 29.44 mA cm^−2^, and interesting *V*_on_ of 0.73 *V*_RHE_, representing a significant advancement in PEC photocathodes. These record results not only demonstrate the potential of CZTS-based photocathodes in PEC water splitting for sustainable hydrogen production, but also underscore the significance of the persistent study in this field to further enhance its practical applications in renewable energy technologies. The video recorded under AM 1.5G sunlight irradiation in 0.5 M H_2_SO_4_ electrolyte during the operation showing more than 5 h continues hydrogen production (Movie S1), further strengthening remarkable efficiency and long-term stability. Furthermore, the exceptional stability of the CZTS-based photocathode in a 0.5 M H_2_SO_4_ electrolyte, demonstrating over 10 h of operation with only an 11% decrease in current density (Fig. S4), is primarily attributed to the innovative PSLE technique. The PSLE process optimizes grain growth and enhances charge carrier dynamics, resulting in a highly robust and corrosion-resistant structure, even under highly acidic conditions.

### Charge Carrier Dynamics and Analysis of Device Performance Improvement Mechanisms

**Fig. 2:**
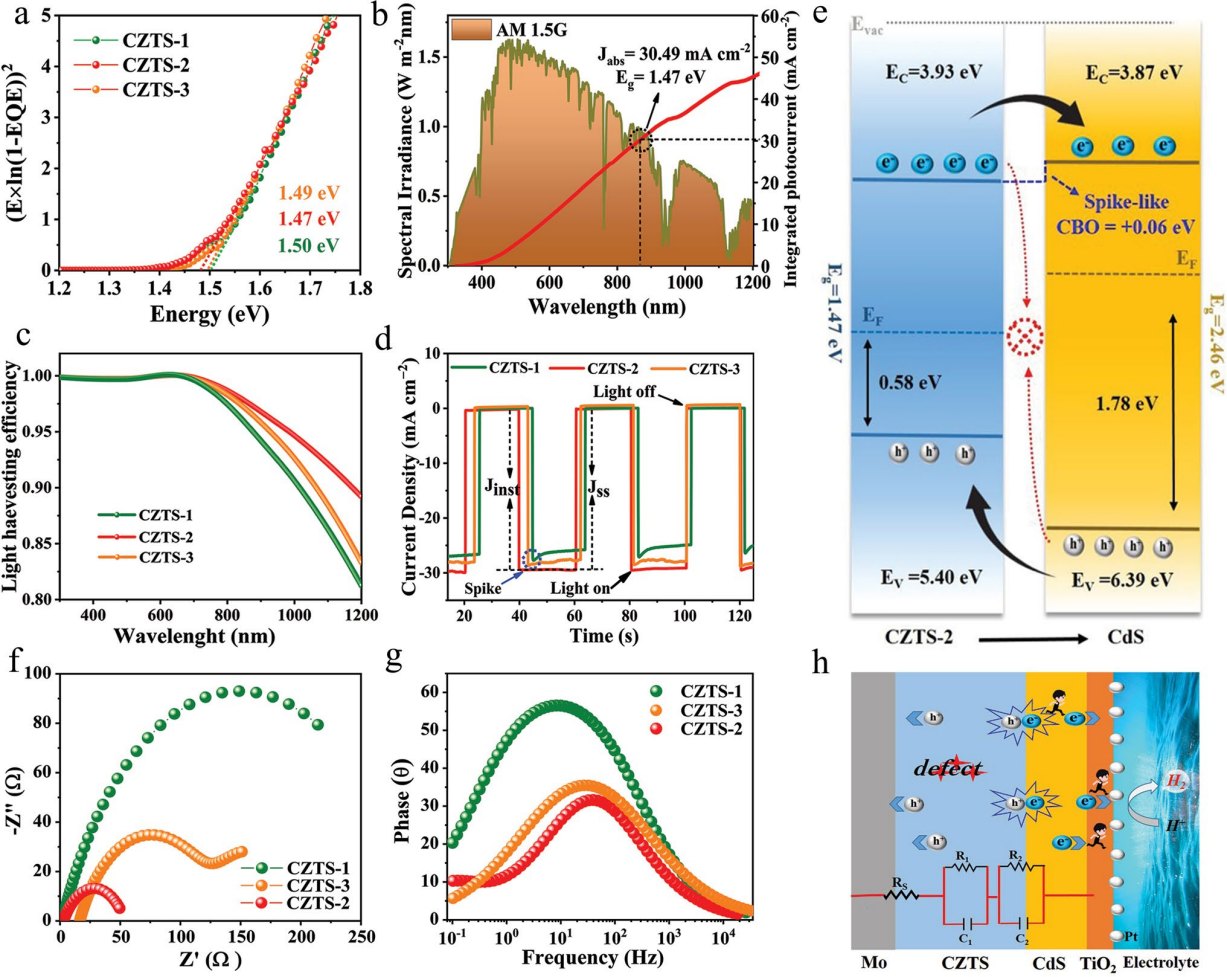
CZTS/CdS heterojunction information and related schematics. **a** (E × ln(1-EQE))^2^ versus energy-based band gap calculation of CZTS-1, CZTS-2, and CZTS-3 thin films. **b** Energy density flux for the standard AM 1.5G solar spectrum, along with the integrated photocurrent density of the CZTS-2. **c** Light harvesting efficiency (LHE). **d** Transient photocurrent response of CZTS-1, CZTS-2, and CZTS-3 photocathodes. **e** Schematic illustration of energy band alignment of CZTS-2/CdS heterojunction. **f** Nyquist plots and **g** the corresponding Bode plots of the CZTS-based photocathodes. **h** Schematic diagram and equivalent circuit system associated with the charge transfer processes

The external quantum efficiency (EQE) spectra of the isostructural CZTS-based photovoltaic (PV) devices were used to derive the band gap (*E*_g_) of the CZTS thin films (Fig. 2a). The *E*_g_ values of the CZTS-1, CZTS-2, and CZTS-3 light-absorbing thin films were measured to be 1.50, 1.47, and 1.49 eV, respectively, falling within the theoretical band gap range of CZTS. The findings also indicate that the *E*_g_ of CZTS can be affected by PSLE, and the narrower band gap exhibited by CZTS-2 offers significant advantages for more efficient sunlight absorption [16]. The results are also consistent with the values obtained from reflectance, transmittance, and absorbance spectra inside wavelength range of 300 to 1200 nm, as presented in Fig. S5c, d. Additionally, the absorption coefficient “*α*”, across the similar wavelength range, was calculated based on absorbance (A) by using $\alpha = \frac{{2.303{\text{A}}}}{{\text{d}}}$ equation [33], further confirming an important *E*_g_ evolution (Fig. S5a, b). Efficient solar harvesting can be encouraged by such a perfect small band gap. Furthermore, the theoretical photocurrent density by using the standard solar spectrum AM 1.5G and the light harvesting efficiency (LHE) of CZTS is determined based on the following formulas [35]:

3 $$\left\{ {\begin{array}{*{20}c} {\begin{array}{*{20}c} {LHE = 1 - 10^{{ - {\text{A}}\left( {\uplambda } \right)}} } \\ {J_{{{\text{ph }} = { }}} J_{{{\text{abs}}}} \times \eta_{{{\text{sep}}}} \times \eta_{{{\text{trans}}}} } \\ {J_{{{\text{abs}}}} = \mathop \smallint \limits_{300}^{{{\uplambda }_{{\text{e}}} }} \frac{{\uplambda }}{1240} \times N_{{{\text{ph}}}} \left( \lambda \right) \times {\text{LHE}}\left( \lambda \right){\text{d}}\lambda } \\ \end{array} } \\ \end{array} } \right.$$

where *J*_abs_ represents theoretically measured photocurrent density, *λ*_e_ is the wavelength at which the band gap is connected to the absorption cutoff and λ is the wavelength, photo flux is represented by *N*_ph_ (λ), and *A* (λ) is the wavelength-related absorbance. The value of *J*_abs_ for CZTS-2 is 30.49 mA cm^−2^ since the *λ*_e_ is ≈844 nm (Fig. 2b). Moreover, absorbance-dependent calculated LHE is presented in Fig. 2c.

The PEC performance of the CZTS-based photocathodes is strongly affected by bulk and interface charge carrier dynamic characteristics. Therefore, as a first step in light of PSLE treatment, an investigation was conducted to view the modification of band alignment to gain a deeper understanding of charge carrier separation, transport, and recombination. Ultraviolet photoelectron spectroscopy (UPS) is employed to analyze the energy band information of CZTS-1, CZTS-2, and CZTS-3 absorbers, as well as the CdS buffer layer, as illustrated in Fig. S6. The CdS and CZTS exhibited a stronger n-type and p-type conductive characteristics. The valence-band offset (VBO) of CZTS-1, CZTS-2, and CZTS-3 for n-CdS/p-CZTS was determined to be in the range of 0.99 to 1.11 eV, which is comparable to previous reports showing VBO of CZTS around 1.14 (± 0.10) eV [36], contributing to a desirable energy barrier that effectively prevents the transfer of photo-generated holes in the valence band of CZTS and inhibits recombination at the electrode–electrolyte interface. In contrast, the conduction-band offset (CBO), calculated from electron affinity (χ) differences, for CZTS-1, CZTS-2, and CZTS-3 thin films is − 0.07, + 0.06, and − 0.06 eV, respectively. Negative CBO values at CZTS-1/CdS and CZTS-3/CdS interfaces indicate “cliff-like” band alignment at their heterojunction interface (Fig. S7). Prior research has suggested that cliff can serve as a barrier against injected electrons under forward bias conditions, potentially leading to an increased risk of charge carrier recombination [34, 37], and would intrinsically diminish band bending and built-in voltage (*V*_bi_) and restrict device performance [32]. Conversely, CZTS-2/CdS exhibits a “spike-like” alignment (positive CBO: + 0.06 eV), within the optimal 0–0.4 eV range for heterojunctions (Fig. 2e). This configuration minimizes carrier accumulation at the interface, promotes efficient charge separation and transport to the electrolyte interface, and enhances overall PEC performance of the CZTS-2 device [38].

In light of the above findings, Mott–Schottky (M-S) measurements were conducted to evaluate the capacitance at the semiconductor–electrolyte interface as a function of the applied voltage (V) (Fig. S8). These measurements provide critical insights into the flat-band potential and carrier density, further elucidating the electronic properties and charge transfer behavior of the CZTS-based photocathode. Near the space charge region (SCR), the reciprocal of the square of the capacitance (1/C^2^) decreases with increasing potential V. The observed behavior demonstrates a distinct p-type characteristic for the CZTS film. The carrier density (*N*_D_) and flat-band potential (${E}_{\text{fb}}$) were determined using the following equations [39]:

4 $$\left\{ {\begin{array}{*{20}c} {N_{{\text{D}}} = \left( {\frac{2}{{q\varepsilon \varepsilon_{0} }}} \right)\left[ {\frac{{{\text{d}}\left( {\frac{1}{{C^{2} }}} \right)}}{{{\text{d}}V}}} \right]^{ - 1} } \\ { \frac{1}{{C_{{{\text{sc}}}}^{2} }} = \frac{2}{{q\varepsilon \varepsilon_{0} N_{{\text{D}}} }}\left[ {\left( {V - E_{{{\text{fb}}}} } \right) - \frac{{k_{{\text{B}}} T_{{\text{a}}} }}{q}} \right]} \\ \end{array} } \right.$$

where *C*_SC_ denotes for the SCR capacitance that forms at the semiconductor–electrolyte interface and *q*, *ε*, *ε*_0,_ *k*, and *T*_a_ are the elementary charge, relative permittivity, permittivity of free space, Boltzmann constant, and the absolute temperature, respectively. The analysis reveals that the acceptor density *N*_D_ of the CZTS-2 thin film (3.18 × 10^16^ cm^−3^) is substantially lower than that of CZTS-1 (2.27 × 10^17^ cm^−3^) and even CZTS-3 (9.34 × 10^16^ cm^−3^). It suggests that there are fewer acceptor defects, such as Cu_Sn_ antisites in the CZTS-2 thin film [5]. The PSLE-dependent charge carrier transfer kinetics modification was also investigated. Figure 2d shows the transient photocurrent decay spectra of CZTS-1, CZTS-2, and CZTS-3 photocathodes, with corresponding *J*_ph_ of 26.50, 29.44, and 28.19 mA cm^−2^ in the “light on” state at 0 *V*_RHE_. A distinct “spike-like” transient behavior is evident in both CZTS-1 and CZTS-3 photocathodes, indicating temporary charge accumulation at surface and/or interface states. A larger “spike-like” transient means more electrons are being trapped, indicating poorer charge transfer efficiency (*η*_tran_) and separation efficiency (*η*_sep_) in the PEC system. The captured photoelectrons in these states tend to recombine with holes before reaching a stable state due to a significant energy barrier. Comparatively, no transient spike observed for CZTS-2 suggests reduced carrier recombination. In parallel, the *η*_sep_ values were calculated and for the champion CZTS-2 device (99.6%) is significantly higher than CZTS-1 (93.1%) and CZTS-3 (97.7%) as shown in Fig. S9, which illustrate that the CZTS-2 photocathode is far more efficient in promoting charge carrier separation and transport, which is strongly linked to its much superior interface characters with benign band alignment, enhanced electron density, and reduced defect density due to PSLE treatment. Hence, in the case of the CZTS-2 photocathode, the charge recombination can be deemed negligibly minimal, leading to larger *η*_sep_, which contributes to our achievement of a *J*_ph_ of 29.44 mA cm^−2^, closely approaching theoretical value of 30.49 mA cm^−2^, and as a result, record STH efficiency of 9.91%.

Moreover, PEIS serves as a valuable strategy for assessing charge transfer resistance, was applied under illumination, and is shown in Fig. 2f–h. Figures 2f and S10 depict the Nyquist plots of the CZTS-1, CZTS-2, and CZTS-3 photocathodes. The PEIS spectra display semicircles of different diameters, with smaller diameters suggesting lower resistance at the electrified interface [40], a finding that aligns with results obtained by the transient response of the three devices. Three distinct peaks can be seen in the corresponding Bode diagrams, which are located at low frequencies (10^–1^–10^1^ Hz) and high frequencies (~ 10^1^–10^4^ Hz) (Fig. 2g). An analogous circuit (Fig. 2h) was used to fit the PEIS results with each model included two sets of resistor–capacitor (R–C) components and one series resistance (*R*_s_). The *R*_s_ series resistance value is attributed to Mo/CZTS back contact at the interface. The associated charge transport resistance and the internal defect capacitance and resistance in CZTS films are represented by *C*_1_ and *R*_1_ (high frequency derivatives). In parallel, the low-frequency arc derivatives *R*_2_ and *C*_2_ denote the resistance of electrochemical charge transport and response inside the Helmholtz layer at the photocathode–electrolyte interface and the capacitance associated with surface/interface states, respectively [41].

Based on the fitted results (Table S3), the values of *R*_1_ and *R*_2_ for the CZTS-2 photocathode are significantly smaller than those observed for the CZTS-1 and CZTS-3 photocathodes. This discrepancy suggests to more effective HER mechanisms at the electrode–electrolyte interface in the CZTS-2 photocathode, as well as more effective separation and transport of charge carriers. Additionally, a smaller *R*_1_ value of 47.87 Ω indicates fewer bulk defects within the CZTS-2 absorber as compared to CZTS-1 (270.8 Ω) and CZTS-3 (123.2 Ω). Meanwhile, a smaller *R*_2_ value also suggests enhanced carrier separation and transport at the interface. Importantly, the exceptionally smaller *C*_2_ value of CZTS-2 (5.22 × 10^–5^ F) photocathode indicates a suppressed interface recombination. Furthermore, the highest series resistance *R*_s_ observed at the Mo/CZTS-3 back contact interface can be attributed to the increased roughness and the presence of voids resulting from excessive PSLE treatment, in agreement with SEM observations.

### Microstructure Characterizations and Evaluations

**Fig. 3:**
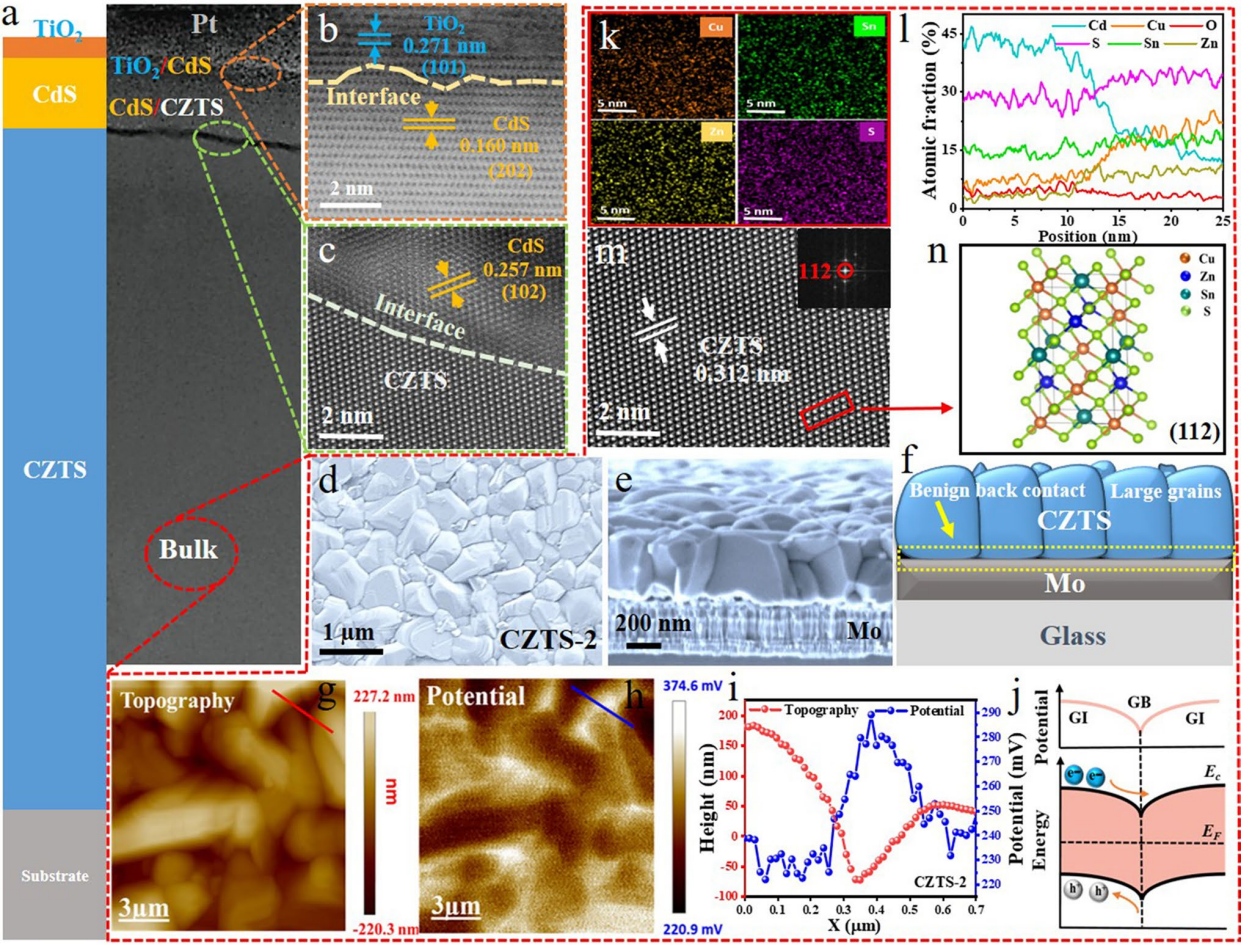
Detailed microstructure investigation of Mo/CZTS/CdS/TiO_2_/Pt photocathode. **a** Cross-sectional TEM image of CZTS-2-based photocathode. High-resolution TEM images of **b** CdS/TiO_2_ and **c** CZTS/CdS interfaces. SEM images of CZTS-2 absorber layer, **d** surface, **e** cross section, and **f** schematic illustration of cross section. **g** KPFM scanning surface topography, **h** potential, **i** CPD maps of CZTS-2 thin film, and **j** schematic diagrams of the energy band structure and CPD near the GBs for CZTS-2 thin films under the PSLE conditions. **k** TEM-coupled EDS elemental mappings of Cu, Zn, Sn, S, and Cd. **l** TEM-EDS elemental line scan profiles across CZTS-2. **m** High-resolution TEM images of CZTS-2 bulk. **n** Its crystal lattice diagrams

Through systematic optimization of the hot-plate annealing duration, a thermodynamic nucleation evolution was deeply observed in the CZTS precursor thin films. This evolution led to a transformation in film morphology, transitioning from clustered-like structures to well-separated granular structures. Hence, the systematic structural and morphological analyses, comparing systematically annealed CZTS precursor films (CZTS-1, CZTS-2, CZTS-3), reveal that annealing duration critically governs interfacial energy redistribution, thereby dictating nucleation density and grain-boundary mobility as shown in Figs. 3 and S11–S15. TEM and high-resolution TEM (HRTEM) characterization were used to investigate the microstructure of the champion Mo/CZTS/CdS/TiO_2_/Pt photocathode. The multilayer structure is clearly visible in the cross-sectional TEM images, showing distinct interfaces between the CZTS/CdS and CdS/TiO_2_ interface layers having spin-coated CZTS light absorber thin films, CBD deposited CdS, and ALD deposited TiO_2_ layers of 1.00 µm, 80 nm, and 10 nm thicknesses, respectively (Fig. 3a). Notably, increasing annealing duration induced a marginal thickness reduction in CZTS precursor films, decreasing from ~ 1.356 µm (100 s) to 1.335 µm (200 s) (Fig. S13), attributed to progressive film densification. After post-sulfurization at 620 °C, all films converged uniformly to ~ 1.00 µm, eliminating initial variations. Furthermore, the distance of the (112) CZTS lattice plane is matched by the lattice fringes shown in the HRTEM picture with an inter-planar spacing of 0.312 nm (Fig. 3m). The observed lattice stripe had an inter-planar spacing of about 0.271 nm, which corresponds to (101) lattice plane of TiO_2_ (PDF No. 21–1276), and a CdS buffer layer having inter-planer spacing of 0.160 nm corresponding to its (202) lattice plane (PDF No. 01-080-0006) (Fig. 3b). HRTEM images of the CZTS/CdS interface show a smooth and well-adherent interface without any voids (Fig. S11). The lattice structures of these two materials are matched, which can help reduce current leakage and recombination losses [32]. The CdS buffer layer near the CZTS/CdS interface exhibits an inter-planar spacing of 0.257 nm, corresponding to the distance between the (102) lattice planes (Fig. 3c). TEM and HRTEM analyses reveal that the optimized PSLE results in a high-quality CZTS-2 thin film without the formation of bulk or cross-sectional cracks or pinholes. XRD investigations further corroborate this observation. The synthesized CZTS thin films exhibit a typical kesterite crystal structure and high crystallinity, as demonstrated by the Raman spectroscopy and XRD analysis conducted both before and after post-sulfurization (Fig. S12) [42]. SEM analyses clearly show transformation in CZTS-1, CZTS-2, and CZTS-3 precursor film morphology from clustered structures to well-separated granular structures under the PSLE treatment and significant influence of this transformation on the nucleation, growth, and recrystallization processes during sulfurization (Fig. S14). Specifically, CZTS-1 and CZTS-3 thin film primarily consists of non-uniform clusters. Upon sulfurization, recrystallization takes place, resulting in the emergence of distinct pinholes and random grain structures in CZTS-1 and CZTS-3 thin films. In contrast, recrystallization of CZTS-2 thin films shows dense and uniformly larger grain sizes (Fig. 3d), as well as benign back contacts with Mo substrate (Fig. 3e, f). Additionally, the SEM analyses of CZTS thin films prepared at 50, 250, 300, and 350 s of annealing duration were also conducted and show similar trends as shown in Fig. S15. Moreover, in comparison with CZTS-1 and CZTS-3, the CZTS-2 thin film shows reduced full width at half maximum (FWHM), consequently larger grain sizes (Fig. S16), echoing a higher degree of crystallinity in CZTS-2 under PSLE-induced kinetic grain growth. The findings of line scanning and element mapping using TEM-coupled EDS demonstrate the uniform and sensible distribution of chemical elements in the CZTS absorber layer (Fig. 3k, l). Figure S17 shows the XPS spectra of CZTS thin films exhibit well-defined peaks, confirming the high chemical purity and stoichiometric composition of the films. Distinct peaks corresponding to Cu 2*p*, Zn 2*p*, Sn 3*p*, and S 2*p*, 2*s* core levels were observed, with binding energies consistent with the expected oxidation states of Cu⁺, Zn^2^⁺, Sn^4^⁺, and S^2^⁻, respectively. The absence of secondary phases or impurity-related peaks in the spectra highlights the effectiveness of the PSLE method in producing phase-pure CZTS thin films. Finally, the surface topography and surface potential of the optimized CZTS-2 (Fig. 3g–j), as well as the CZTS-1 and CZTS-3 thin films (Fig. S18), were characterized using Kelvin probe force microscopy (KPFM). Surface topography demonstrates SEM-aligned morphology, while the contact potential difference (CPD) potential mapping (Fig. S19) demonstrates synchronous variation with the topography. It is evident that, in the majority of the locations, brightness at grain borders (GBs) was comparatively greater than that in grain internals (GIs), and that GB brightness increased steadily from CZTS-1 to CZTS-3. Therefore, it is advantageous for there to be a downward band bending at GBs in order to draw electrons and repel holes and promote electron–hole separation [43]. The average potential difference between GBs and GIs was approximately 27, 42, and 62 mV for CZTS-1, CZTS-2, and CZTS-3, respectively, suggesting increasing band bending at GBs from CZTS-1 to CZTS-2 to CZTS-3. The schematic band diagrams depicted in Fig. S18d, h, l show the progression of band bending. Moreover, based on the work function of the scanning probe tip ($\varphi_{t}$) and the relative contact potential difference (*V*_CPD_), the work function of the sample ($\varphi_{s}$) can be determined using the following equation:

5 $$\varphi_{s} = \varphi_{t } + {\text{e}}V_{{{\text{CPD}}}}$$

From the obtained results presented in Figs. 3g-j and S17, it can be observed that the sample CPD increases with decreasing surface height from grain interiors (GIs) to grain boundaries (GBs). Accordingly, $\varphi_{s}$ at GB is higher than that at GI, while keeping $\varphi_{t}$ constant. The favorable CPD value for CZTS-2 as compared to CZTS-1 and CZTS-3 denotes the upshift of the Fermi level, which indicates that the 150 s of annealing treatment has favorably increased the carrier concentration. The higher Fermi level would result in relatively larger downward band bending, hindering the hole at the front interface and leading to a larger *V*_on_ as well. According to the literature, regarding the occurrence of defect trapping, an appropriate upshift in the fermi level would facilitate the separation and collection of electrons and holes, and therefore reducing the carrier recombination [44]. Overall, the optimized CZTS-2 film with large and dense crystals, suitable thickness, and benign band bending is obtained, which promotes the light absorption and favorable for the generation, separation and transport of the charge carrier, and therefore higher *V*_on_, *J*_ph_, and HC-STH efficiency in CZTS-2 photocathode [45].

### Defect Dynamics

**Fig. 4:**
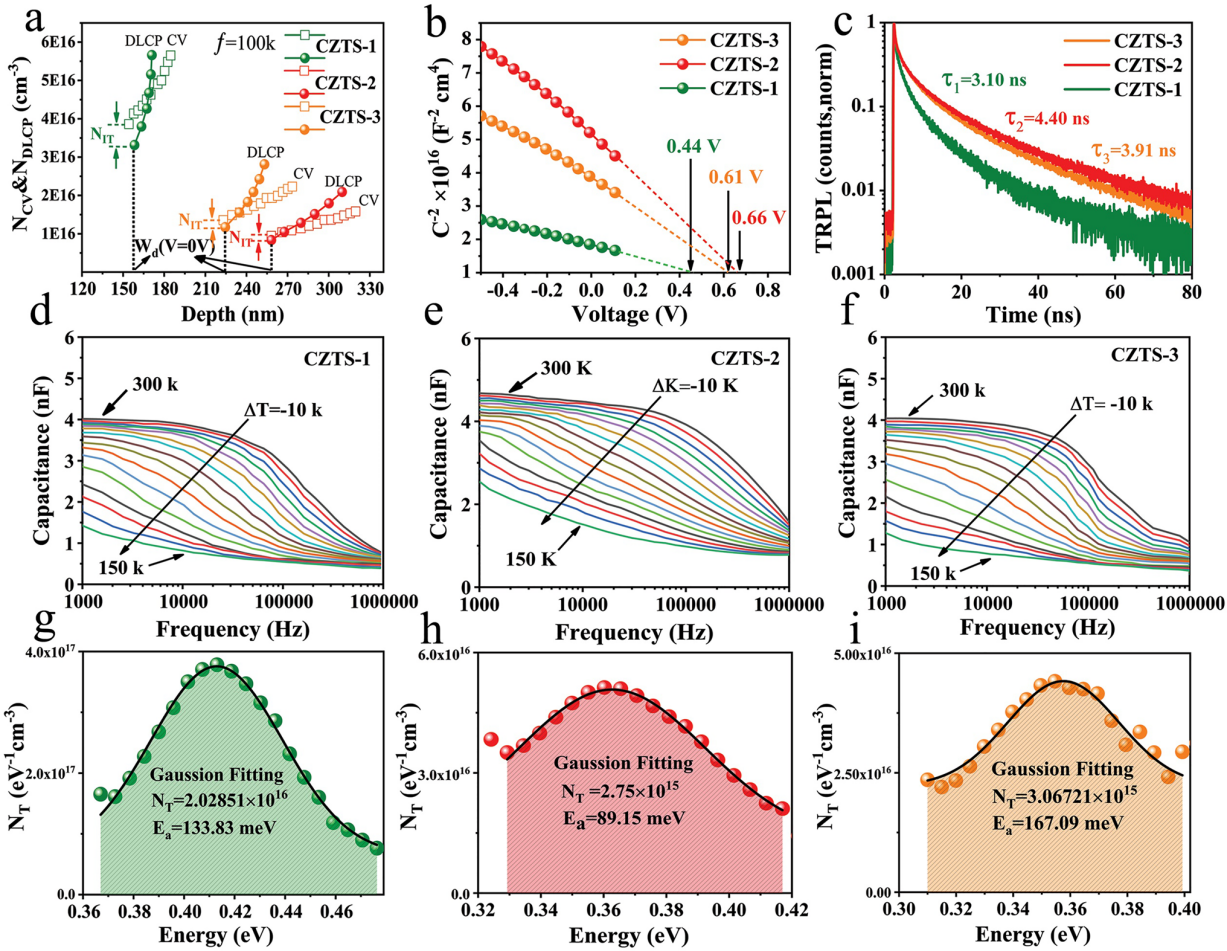
Characterization of bulk and interface defects and charge carrier dynamics in CZTS-based devices. **a** C-V and DLCP profiles. **b** 1/C^2^-V plots. **c** TRPL characterizations of the corresponding CZTS-1, CZTS-2, and CZTS-3 thin films. **d-f** Temperature-dependent admittance characterizations, capacitance variation at different frequencies and temperatures of CZTS-1, CZTS-2, and CZTS-3 PV devices. **g-i** The corresponding representations of defect distributions

It is expected that improvements in *J*_ph_ and HC-STH conversion efficiency can also be ascribed to the suppressed defect density in the PSLE-treated CZTS thin-film photocathode. To evaluate this influence, capacitance–voltage (*C-V*) and deep-level capacitance profiling (DLCP) measurements were performed, and the outcomes are displayed in Fig. 4a, b. In general, C-V measurements assess the capacitance responses, which include bulk defects, free carriers, and interface defects. On the other hand, DLCP measurements correspond to the responses of free carriers and bulk defects. The following formulas are used to get the *N*_C-V_ and *N*_DLCP_ plots versus the profiling depth $x$ [46]:

6 $$\left\{ {\begin{array}{*{20}c} {N_{C - V} = \frac{{ - 2\varepsilon_{r,n} N_{D} }}{{\left( {\frac{{{\text{d}}\left( {\frac{1}{{C^{2} }}} \right)}}{{{\text{d}}V}}} \right)qS^{2} \varepsilon_{0} \varepsilon_{r} \varepsilon_{r,p} N_{D} + 2\varepsilon_{r,n} }}} \\ {N_{{{\text{DLCP}}}} = - \frac{{C_{0}^{3} }}{{2q\varepsilon_{0} \varepsilon_{r,p} S^{2} C_{1} }}} \\ {x = \frac{{2\varepsilon_{0} \varepsilon_{r,p} S}}{{C_{0} }}} \\ \end{array} } \right.$$

where *N*_D_, *A*, *ε*_r,n_, *ε*_r,p_, *C*_0_, and *C*_1_ are the CdS doping density, the device area, the CdS and CZTS relative permittivity, and two parameters for quadratic fitting obtained from the C-V curves, respectively. Thus, the interface defect density (*N*_IT_) for CZTS-2 device is calculated to be 9.88 × 10^15^ cm^−3^ (Fig. 4a), which is notably lower than the CZTS-1 and CZTS-3 devices (Table 1). This suggests that the PSLE treatment effectively inhibits interface defects and defect-assisted recombination, leading to improved charge carrier dynamics and enhanced PEC performance in case of CZTS-2 photocathode. Furthermore, the heterojunction depletion width (*W*_d_) of the CZTS-1, CZTS-2, and CZTS-3 devices was determined to be 157, 258, and 224 nm, respectively. Owing to its comparatively larger *W*_d_, CZTS-2 is expected to exhibit enhanced photo-carrier extraction and transport [5]. Figure 4b illustrates the 1/C^2^-V curves, which were used to determine the *V*_bi_ values through linear fitting and extrapolation to the x-axis. The *V*_bi_ at the CZTS-2/CdS heterojunction (0.66 V) was found to be higher than those at the CZTS-1/CdS (0.44 V) and CZTS-3/CdS (0.61 V) configurations. This indicates an enhanced quality of the CZTS-2/CdS heterojunction, which could facilitate photo-generated carrier separation, improve the device’s *V*_on_, and ultimately lead to higher PEC performance. Time-resolved photoluminescence (TRPL) analysis was conducted to determine the minority carrier lifetimes (Fig. 4c). As illustrated, the τ_1_, τ_2_, and τ_3_ values associated with CZTS-1, CZTS-2, and CZTS-3 thin films were found to be 3.10, 4.40, and 3.91 ns, respectively. The minority carriers with a longer lifetime are expected to have a higher likelihood of migrating toward the electrode/electrolyte interface to contribute in HER and consequently results in higher PEC performance. Additionally, in support of these observations, the minority carrier lifetime for the CZTS-0 thin film has been measured at 0.24 ns, and the photoluminescence (PL) spectroscopy results of three devices are collectively displayed in Fig. S20. The CV-DLCP and TRPL analyses of CZTS-1, CZTS-2, and CZTS-3, along with their comparison, clearly demonstrate that the PSLE technique is an effective strategy for mitigating defects in both the CZTS-2 bulk and the CZTS-2/CdS heterojunction. This reduction in defects enhances charge carrier separation and transport toward the electrode–electrolyte interface, enabling greater participation in HER reactions. Subsequently, temperature-dependent admittance spectra (AS) of CZTS-1, CZTS-2, and CZTS-3 were then acquired in order to gain a better understanding of the distribution of defects across the bulk absorber layer. The related photovoltaic (PV) devices were constructed and designated as PV-CZTS-1, PV-CZTS-2, and PV-CZTS-3, respectively, with identical device configurations (e.g., Mo/CZTS/CdS/ITO/Ag). The temperature-dependent capacitance–frequency (C-f-T) plots are displayed in Fig. 4d–f. The modulation frequencies (ω_0_ = 2πƒ_0_) were obtained by plotting the angular frequency point ω at the maximum value of ωdC/dω. The slops of a linear fit of the Arrhenius plots were used to determine the defect activation energies based on the following equation [47]:

7 $$\ln \left( {\frac{{\omega_{o} }}{{T^{2} }}} \right) = \ln \left( {2v_{o} } \right) - \frac{{E_{A} }}{{K_{B} T}}$$

where ${v}_{0}$ represents the attempt-to-escape frequency, and *E*_A_ denotes the mean defect energy-level to either the conduction-band edge or valence-band edge. In CZTS thin films, at each phase, the transition frequency and the proportion of carrier trapping and release from key bulk defect states are correlated [48]. Naturally, when the temperature rises, they will change to a higher frequency. The energy gap for the occupancy of hole traps in SCR for a p-type CZTS semiconductor is thus ${E}_{\text{a}}={E}_{\text{T}}-{E}_{\text{V}}$ above the valence band, where ${E}_{\text{a}}$, ${E}_{\text{T}}\text{ and}{ E}_{\text{V}}$ are activation, trap, and valence energies, respectively. Figure S21 shows the Arrhenius plots of the transition frequencies, from which ${E}_{a}$ values can be obtained by fitting. After superimposing the differential capacitance spectra at individually temperature, an energy defect distribution (${N}_{\text{T}}$ (${E}_{\text{w}}$)) is obtained according to the following equations [49]:

8 $$E\left( \omega \right) = kT \ln \left( {\frac{{2\pi v_{o} T^{2} }}{\omega }} \right)$$

9 $$N_{T} \left( {E_{\omega } } \right) = - \frac{{V_{bi} }}{qWkT}\frac{\omega dC}{{d\omega }}$$

where ω is the applied frequency and ${v}_{o}$ is the emission factor. Previous studies and the theoretical foundation of first principle [50] indicate that the copper vacancy (*V*_Cu_) has a higher formation energy than the Cu_Zn_ antisite defect. The acceptor defect with ${E}_{\text{a}}$ in the range of 0.13–0.2 eV can be attributed to the Cu_Zn_ antisite defect, whereas a shallower acceptor level at ≈0.05 eV above the valence-band maximum (VBM) is assumed to represent *V*_Cu_. Therefore, *V*_Cu_ may thus be ascribed to the defect energy distribution in device PV-CZTS-2, with a peak value centered at 0.0895 eV. On the other hand, the values in devices PV-CZTS-1(0.133 eV) and PV-CZTS-3 (0.167 eV) can be attributed to the Cu_Zn_ antisite. It has been proven that in order to achieve high-performance photoelectric devices, *V*_Cu_ with a shallow acceptor level is far superior than Cu_Zn_ antisite [49]. Furthermore, in fitting defect states as a Gaussian distribution the integrated defect density functions (*N*_T_) of the PV-CZTS-1, PV-CZTS-2, and PV-CZTS-3 devices are presented in Fig. 4g–i, and their values are 2.02 × 10^16^, 2.75 × 10^15^, and 3.06 × 10^15^ cm^−3^, respectively. The variation in defect density values in the absorber layer shows efficient bulk defect passivation and non-radiative recombination PV-CZTS-2 device. Overall, as summarized in Table 1, CZTS-2, through appropriate PSLE treatment, exhibits modified defect characteristics in both the bulk and interface regions. This modification is beneficial for suppressing defect-assisted charge carrier recombination, improving minority carrier lifetime, and enhancing carrier separation and transfer efficiencies. As a result, the CZTS-2 photocathode achieves a record HC-STH conversion efficiency of 9.91% and a current density of 29.44 mA cm^−2^.

**Table 1:** A summary of PSLE-derived PEC performance and charge carrier dynamic parameters of the CZTS-1, CZTS-2, and CZTS-3 photocathodes

Device	*J*_ph_ (mA cm^−2^)	*V*_on_ (V_RHE_)	HC-STH (%)	*N*_IT_ (cm^−3^)	*N*_T_ (cm^−3^)	*τ* ns	*V*_bi_ (V)	*W*_d_ (nm)
CZTS-1	26.50	0.70	8.21	3.87 × 10^16^	2.02 × 10^16^	3.10	0.44	157
CZTS-2	29.44	0.73	9.91	9.88 × 10^15^	2.75 × 10^15^	4.40	0.66	258
CZTS-3	28.19	0.71	8.82	1.34 × 10^16^	3.06 × 10^15^	3.91	0.61	224

### Applications Demonstration of Scalable CZTS-Based Photocathode and Unbiased Cu_2_ZnSnS_4_-BiVO_4_ Tandem Cell in Natural Seawater

**Fig. 5:**
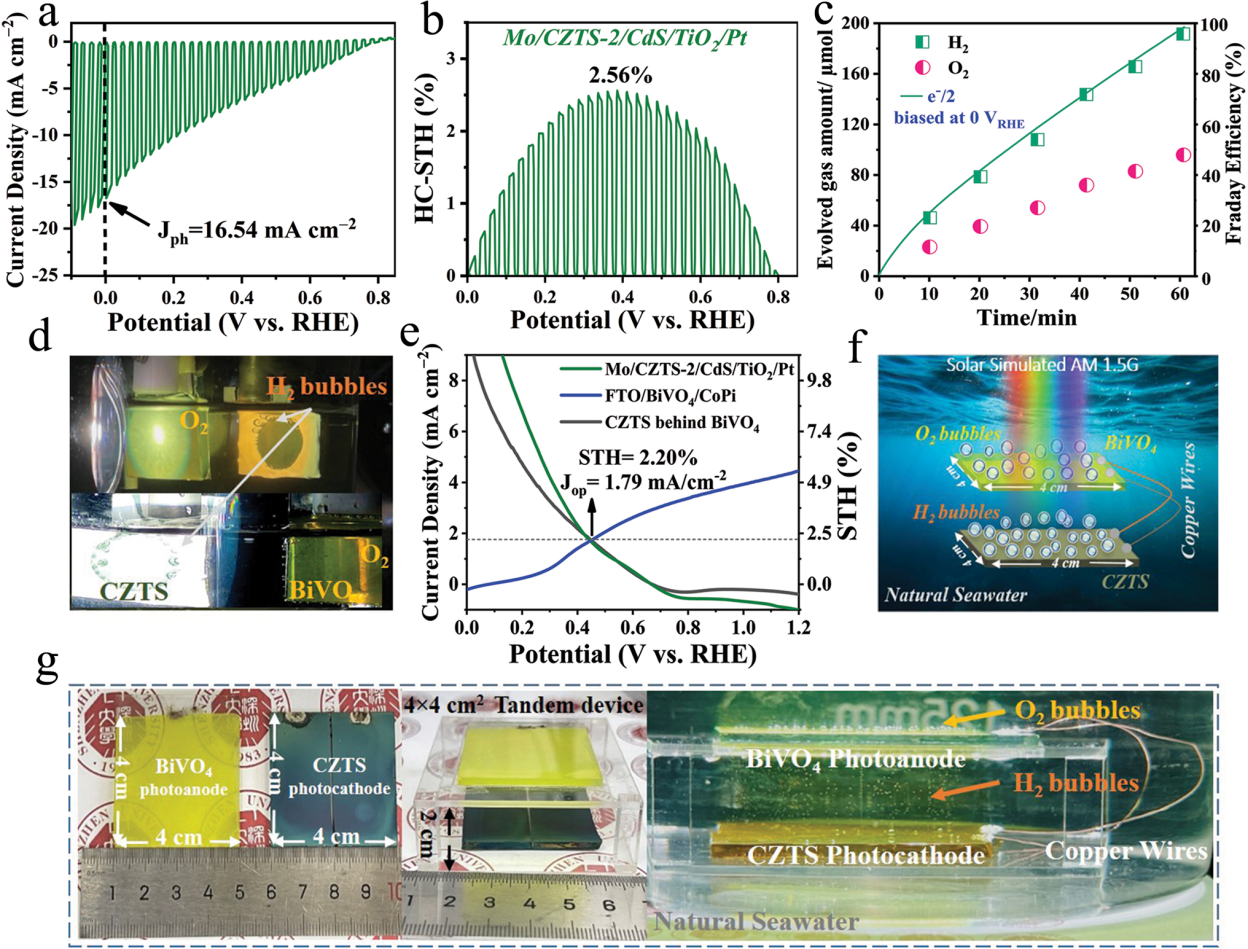
PEC performance of Mo/CZTS-2/CdS/TiO_2_/Pt photocathode and scalable CZTS-BiVO_4_ tandem device under solar simulated AM 1.5G irradiation in seawater, pH 6.5 (± 0.2). **a** Chopped J-V curve of Mo/CZTS-2/CdS/TiO_2_/Pt photocathode. **b** Corresponding calculated HC-STH conversion efficiency. **c** Hydrogen and oxygen evolution amount-time curves. **d** Captured photographs of CZTS-BiVO_4_ tandem cell configuration. **e** Obtained J-V curves of the CZTS photocathode, BiVO_4_ photoanode, and CZTS photocathode behind the BiVO_4_ photoanode. **f** Schematic illustration of 4 × 4 cm^2^ CZTS-BiVO_4_ tandem device.** g** Photographs and working of as prepared 4 × 4 cm^2^ CZTS-BiVO_4_ tandem device and its seawater splitting operation at unbiased voltage

Recognizing that achieving high PEC performance in natural electrolytes such as seawater poses a significant challenge for CZTS-based photocathodes, due to high chloride ion concentrations, dissolved oxygen, and the potential for multiple corrosion mechanisms, this study represents the first demonstration of the potential of CZTS-based photocathodes for solar-driven seawater splitting. While conventional electrolytes are ideal for fundamental studies, seawater presents a more realistic yet challenging pathway for scalable hydrogen production due to its harsh conditions. By leveraging seawater as an abundant, cost-effective, and eco-friendly resource, we aim to advance scalable and economically viable PEC technologies, paving the way for their integration into real-world energy systems and sustainable hydrogen production. To this end, we evaluated the PEC properties of PSLE-prepared Mo/CZTS-2/CdS/TiO_2_/Pt devices in natural seawater. The seawater was filtered using a commercially available 0.4 µm filter to remove insoluble salts, organic matter, microorganisms, and other impurities before testing. Our Mo/CZTS-2/CdS/TiO_2_/Pt photocathode demonstrated interesting *J*_ph_ of 16.54 mA cm^−2^ at 0 V_RHE_, and HC-STH efficiency of 2.56% (Fig. 5a, b). Importantly, it is one of the highest recorded PEC properties of CZTS-based photocathodes in seawater and/or neutral electrolytes (Table S4), strengthening the effectiveness of our PSLE-induced grain growth and charge carrier dynamics optimization. The Mo/CZTS-2/CdS/TiO_2_/Pt photocathode was further tested in 0.2 M Na_2_HPO_4_/NaH_2_PO_4_-based electrolytes at pH 4.0 and 7.0 to benchmark its performance against seawater conditions. It achieved HC-STH efficiencies of 4.64% (pH 4.0) and 3.51% (pH 7.0), accompanied by photocurrent densities of 24.19 and 19.83 mA cm^−2^, respectively (Fig. S22). Notably, Mo/CZTS-2/CdS/TiO_2_/Pt photocathode remarkably retained 85% of its initial current density after 5 h of continuous operation in seawater. While this degradation is marginally higher than in controlled pH4 (88% retention) and pH7 (95% retention) environments, the stability remains notable given seawater’s aggressive conditions, as illustrated in Fig. S23. This stability is likely attributed to the intrinsic corrosion resistance of CZTS, optimized morphology and dense, defect-minimized microstructure of our CZTS films synthesized by the strategic implementation of PSLE, which mitigates chloride-induced degradation. As evidenced by SEM and TEM analyses, the CZTS-2 photocathode exhibits a compact crystalline structure free of cracks or voids (Figs. 3 and S14, S15), which physically limits chloride ion penetration and delays bulk corrosion by minimizing pathways for electrolyte infiltration. Furthermore, the low Sn/Cu ratio in our thin film, confirmed by TEM-EDS elemental line scans (Fig. 3i), suppresses the formation of SnS₂ secondary phases [28, 51]. These phases are inherently unstable in saline environments due to their propensity for dissolution, and their absence enhances the material’s resilience. These findings highlight the robustness and potential of CZTS-based photocathodes for practical applications in harsh environments. Subsequently, Fig. 5c illustrates the highly consistent hydrogen (H_2_) and oxygen (O_2_) production from our champion photocathode biased at 0 *V*_RHE_. The gas chromatography detection comprises (1) a reaction chamber evacuated to < 1 Pa to minimize residual gas interference, enabling controlled sample processing and gas generation (e.g., H_2_/O_2_), and (2) a gas chromatograph with a thermal conductivity detector (TCD), which identifies gas species via retention times and quantifies concentrations through signal intensity, calibrated to ensure analytical precision. Consequently, H_2_ and O_2_ gases were generated at constant rates exceeding 190 and 90 µmol cm^−2^ h^−1^, respectively, with a calculated Faradaic efficiency surpassing 95%, collectively representing the highest real-time hydrogen production ever recorded through PEC water splitting. A recording of the test is provided in Movie S2.

It should be noted that our CZTS-based photocathode exhibits very promising solar seawater splitting photovoltage > 0.75 *V*_RHE_ and such a high onset potential is very important and suitable to assemble efficient tandem of photocathode-photoanode cells for standalone devices [6]. Hence, we fabricated the CZTS-BiVO_4_ tandem cell with 0.95 cm^2^-2cm^2^  cm effective area, followed by the development of a larger cell with an active area of 4 × 4 cm^2^ (Fig. 5g). The current–potential curves were obtained from Mo/CZTS-2/CdS/TiO_2_/Pt (0.95 cm^2^) photocathode and FTO/BiVO_4_/CoPi photoanode (2 cm^2^), and thus, a two-electrode tandem cell was assembled by placing a BiVO_4_ photoanode in front of a CZTS photocathode followed by exposure to simulated AM 1.5G solar radiation, as shown in Fig. 5d.

The operational point of working tandem cell is observed at an impressive operating point photocurrent density (*J*_op_) of 1.79 mA cm^−2^ and *V*_on_ of 0.45 *V*_RHE_ (Fig. 5e). Furthermore, the remarkable 2.20% STH efficiency of tandem cell was calculated based on the STH efficiency calculation equation:

10 $$\eta_{{{\text{STH}}}} = \left( {J_{{{\text{op}}}} \times 1.23} \right)/p$$

where *P* is the power of illuminating light. This 2.20% STH efficiency is the highest unbiased STH efficiency for CZTS-BiVO_4_ tandem cells in seawater and/or natural electrolytes. The recorded video with tandem operation is shown in Movie S3. For large-scale practical applications of unbiased hydrogen production, the size of the device plays a crucial role. Although not inherently challenging, due to limitations in our operational setup, we fabricated a 4 × 4 cm^2^ CZTS-BiVO_4_ tandem cell and demonstrated its excellent real-time performance (Fig. 5g). The 4 × 4 cm^2^ tandem cell operation showcased clear and distinct H_2_ and O_2_ bubbles under simulated AM 1.5G solar radiation in seawater, as depicted in Movie S4, which undoubtedly highlighted the bright prospects of CZTS-based photocathodes for practical green hydrogen production application via *in situ* solar seawater splitting.

## Conclusions

In conclusion, we develop an innovative PSLE technique to carefully modify the precursor seed evolution-dependent crystal growth of the CZTS light-absorbing film. A finely tuned PSLE strategy led to synthesize a high-quality CZTS characterized by large, compact, uniform, and vertically aligned grains. Planar-type photocathodes comprising Mo/CZTS/CdS/TiO_2_/Pt were fabricated and examined. The optimized CZTS films exhibited reduced passivation of bulk and interfacial defects, resulting in a superior CZTS/CdS heterojunction characterized by a higher built-in voltage (0.66 V) and lower defect density (9.88 × 10^15^ cm^−3^) at the interface. Benefiting from enhanced charge carrier generation and separation, as well as reduced defect-assisted recombination, the photocathode achieved a record 9.91% HC-STH conversion efficiency and a *J*_ph_ of 29.44 mA cm^−2^ (at 0 *V*_RHE_), approaching its theoretical value. These metrics represent the highest performance reported among CZTS-based photocathodes for solar water splitting, with stable operation over 10 h and only 11% degradation. Moreover, the intriguing onset potential of 0.73 *V*_RHE_ represents one of the notable outcomes among CZTS-based photocathodes. Furthermore, the champion photocathode exhibited an impressive and highly stable *J*_ph_ of 16.54 mA cm^−2^, HC-STH conversion efficiency of 2.56%, and *V*_on_ of 0.78 *V*_RHE_ in seawater environment, followed by a highest STH efficiency of 2.20% for unbiased CZTS-BiVO_4_ tandem cell. Finally, a 4 × 4 cm^2^ large-area CZTS-BiVO_4_ tandem cell module was fabricated for scalable overall solar seawater splitting device applications. These advancements in the CZTS-based photoelectrodes with their practical perspective demonstrated promising potential for the future of PEC processed in situ solar seawater splitting and pave the way for further exploration of Cu-based chalcogenide photoelectrodes.

## Supplementary Information

Below is the link to the electronic supplementary material.

Supplementary file1 (MP4 7381 kb)

Supplementary file2 (MP4 4869 kb)

Supplementary file3 (MP4 2914 kb)

Supplementary file4 (MP4 7202 kb)

Supplementary file5 (DOCX 10511 kb)
