# Supplementary material for: Highest Solar-to-Hydrogen Conversion Efficiency in Cu2ZnSnS4 Photocathodes and Its Directly Unbiased Solar Seawater Splitting
Source: Nanomicro Lett. 2025 May 16;17:257. doi: 10.1007/s40820-025-01755-8 (PMC12084205; doi:10.1007/s40820-025-01755-8)
Supplement: Supplementary file 5 — Supplementary file5 (DOCX 10511 kb) [file 40820_2025_1755_MOESM5_ESM.docx]

Supporting Information for

**Highest Solar-to-Hydrogen Conversion Efficiency in Cu_2_ZnSnS_4_ Photocathodes and Its Directly Unbiased Solar Seawater Splitting**

Muhammad Abbas^1^, Shuo Chen^1^*, Zhidong Li^1^, Muhammad Ishaq^1^, Zhuanghao Zheng^1^, Juguang Hu^1^, Zhenghua Su^1^, Yanbo Li^2^, Liming Ding^3^*, Guangxing Liang^1^*

^1^Institute of Thin Film Physics and Applications, Shenzhen Key Laboratory of Advanced Thin Films and Applications, Key Laboratory of Optoelectronic Devices and Systems of Ministry of Education and Guangdong Province, State Key Laboratory of Radio Frequency Heterogeneous Integration, College of Physics and Optoelectronic Engineering, Shenzhen University, Shenzhen 518060, P. R. China

^2^Institute of Fundamental and Frontier Sciences, University of Electronic Science and Technology of China, Chengdu 610054, P. R. China

^3^School of Chemical Engineering and Light Industry, Guangdong University of Technology, Guangzhou 510006, P. R. China

*****Corresponding authors. E-mail: [chensh@szu.edu.cn](mailto:chensh@szu.edu.cn) (Shuo Chen); [ding@nanoctr.cn](mailto:ding@nanoctr.cn) (Liming Ding); [lgx@szu.edu.cn](mailto:lgx@szu.edu.cn) (Guangxing Liang)

**Note S1 Preparation of BiVO_4_**

**S1.1 Preparation of BiOI Precursor Film**

The BiOI precursor film was electrodeposited on an FTO glass substrate in a three-electrode system, where a Pt-foil was used as the counter electrode and an Ag/AgCl with saturated KCl solution was used as the reference electrode. The BiOI precursor deposition solution was fabricated by dissolving lactic acid (0.03 M), KI (0.4 M), and Bi(NO_3_)_3_.5H_2_O (0.015 M) in deionized water (100 mL), with 1,4-Benzoquinone (0.046 M) in ethanol (40 mL) solution. The pH of the mixed solution was adjusted to 3.5 by adding 0.1 M nitric acid aqueous solution after stirring for 30 min. Initially, a 60 s deposition was conducted at -0.40 V_Ag/AgCl_ to prevent the falling off of BiOI films from the surface of the FTO substrate. After the initial deposition, the BiOI film was obtained at a constant voltage of -0.28 V_Ag/AgCl_ with different deposition durations, then rinsed thoroughly with deionized water and dried in a drying oven.

**S1.2 Preparation of BiVO_4_ Electrode**

The vanadium source solution was prepared by dissolving VO(acac)_2_ (0.5 M) in dimethylsulfoxide (10 mL). The as-prepared BiOI precursor film was dropped into the superfluous vanadium source solution. The electrode was then shifted to a muffle furnace and annealed for about 12 h. The heating rate was 3 ^o^C/min to 120 ^o^C, 0.67 ^o^C/min to 280 ^o^C, and 1.41 ^o^C/min to 450 ^o^C, and then held at 450 ^o^C for 1 h. All the annealing processes ended with furnace cooling. After annealing, the electrodes were immersed in a 1.0 M NaOH solution for 15 min with gentle stirring to wash off the V_2_O_5_ on the BiVO_4_ surface. The prepared BiVO_4_ electrodes were rinsed thoroughly with deionized water and dried in a drying oven.

**S1.3 Photo-Assisted Electrodeposition of CoPi Cocatalyst**

The CoPi co-catalyst was electrodeposited on the BiVO_4_ electrode under AM 1.5 G simulated sunlight by using the three-electrode system containing the solutions of NaH_2_PO_4_ (0.1 M), Na_2_HPO_4_ (0.1 M), Co(NO_3_)_2_.6H_2_O (0.001 M), and deionized water (100 mL). Similarly, an Ag/AgCl with a saturated KCl solution was used as the reference electrode and a Pt-foil as the counter electrode. The deposition voltage and time were -0.20 VAg/AgCl and 90 s, respectively.

**Table S1** A comparison of this work to those of previously reported CZTS-based photocathodes

| **Photocathode** | **Electrolyte** | **Method** | **J_ph_ (mA cm^−2^ at 0 V_RHE_)** | **V_on_ (V_RHE_)** | | **HC-STH (%)** | **Year** | **Refs.** |
| --- | --- | --- | --- | --- | --- | --- | --- | --- |
| **Mo/CZTS/CdS/TiO_2_/Pt** | **0.5 M H_2_SO_4_** | **Spin Coating** | **29.44** | **0.73** | **9.91** | | **2025** | **This work** |
| Mo/CZTS/In_2_S_3_-Pt | 0.25 M KH_2_PO_4_/0.25 M K_2_HPO_4_ | Co-electrodeposition | 6.8 | 0.50 | - | | 2023 | [S1] |
| Mo/CZTS/CdS/ITO/Pt | 1 M K_2_HPO_4_/KH_2_PO_4_ | Spin Coating | 29 | 0.75 | - | | 2023 | [S2] |
| Mo/CZTS-S | 1M Na_2_SO_3_ | Thermal evaporation | 3.0 | 0.20 | 0.31 | | 2022 | [S3] |
| Mo/ACZTS/Ag | Na_2_HPO_4_/ NaH_2_PO_4_ | Spray deposition | 10 | 0.65 | 2.46 | | 2022 | [S4] |
| CZTS/HfO_2_/CdS/HfO_2_/Pt | 0.2M NaH_2_PO_4_/Na_2_HPO_4_ | Spray deposition | 28 | 0.72 | 7.27 | | 2021 | [S5] |
| CZTS/CdS/TiO_2_-NB/Pt | 0.2M NaH_2_PO_4_/Na_2_HPO_4_ | Spray deposition | 17.2 | 0.70 | 3.70 | | 2020 | [S6] |
| Mo/CZTS/CdS/MoS_X_ | 0.2 M Na_2_HPO_4_/NaH_2_PO_4_ | Spray pyrolysis | 18 | 0.60 | 3.0 | | 2019 | [S7] |

**Table S2** A comparison of this work to those of previously reported state of the art chalcogenide-based photocathodes

| **Photocathode** | **Electrolyte** | **J_ph_ (mA cm^−2^ at 0 V_RHE_)** | **V_on_**  **(V_RHE_)** | **HC-STH (%)** | **Year** | **Refs.** |
| --- | --- | --- | --- | --- | --- | --- |
| **Mo/CZTS/CdS/TiO_2_/Pt** | **0.5 M H_2_SO_4_** | **29.44** | **0.73** | **9.91** | **2025** | **This work** |
| Mo/Cu_2_ZnSn(S,Se)_4_/CdS/TiO_2_/Pt | 0.5 M H_2_SO_4_ | 40.40 | 0.50 | 6.47 | 2023 | [S8] |
| SLG/Mo/Sb_2_Se_3_/CdS (In)/Pt | 0.5 M H_2_SO_4_ | 35.70 | 0.54 | 5.60 | 2022 | [S9] |
| Mo/Cu_2_BaSn(S,Se)_4_/CdS/TiO_2_/Pt | 0.5M KH_2_PO4/Na_2_SO_4_ | 5.54 | 0.39 | 0.49 | 2021 | [S10] |
| a-Si(p-i-n)/Fh/Ni | 1M KOH | 15.60 | 0.67 | 4.08 | 2021 | [S11] |
| FTO/Au/Sb_2_Se_3_/PABA/TiO_2_/Pt | 0.1M H_2_SO_4_ | 35 | 0.50 | 4.70 | 2021 | [S12] |
| Mo/(CuInS_2_)0.81(ZnS)0.19/CdS/Pt | 0.5M KPi | 16.70 | 0.84 | 5.60 | 2019 | [S13] |
| Mo/Cu(In,Ga)Se_2_/CdS/Al_2_O_3_/TiO_2_/Pt | 1M HClO_4_ | 28 | 063 | 9.30 | 2018 | [S14] |
| Au/Cu/CdTe/CdS/Pt | 1 M K_2_HPO_4_/KH_2_PO_4_ | 22 | 0.60 | 3.70 | 2017 | [S15] |

**Table S3** Summary of the PEIS fitted parameters

| **Device** | **R_s_(Ω)** | | **R_1_ (Ω)** | **C_1_(F)** | **R_2_ (Ω)** | **C_2_(F)** |
| --- | --- | --- | --- | --- | --- | --- |
| **CZTS-1** | | 3.971 | 270.8 | 2.192×10^-3^ | 25.09 | 3.411×10^-3^ |
| **CZTS-2** | | 4.974 | 47.87 | 3.67×10^-3^ | 67.92 | 5.22×10^-3^ |
| **CZTS-3** | | 16.02 | 123.2 | 7.07×10^-4^ | 90.10 | 4.70×10^-4^ |

**Table S4** A comparison of J_ph_ in this work to those of previously reported CZTS-based photocathodes in seawater or neutral electrolyte

| **Photocathode** | **Electrolyte** | **J_ph_ (mA cm^−2^ at 0 V_RHE_)** | **V_on_**  **(V_RHE_)** | **Year** | **Refs.** |
| --- | --- | --- | --- | --- | --- |
| **Mo/CZTS/CdS/TiO_2_/Pt** | **Seawater** | **16.54** | **0.78** | **2025** | **This work** |
| Mo/CZTS/CdS/In_2_S_3/_Pt | 6.5 | 5 | 0.63 | 2023 | [S16] |
| FTO/2D-CZTS | 8.0 | 0.041 | 0.30 | 2022 | [S17] |
| Mo/CZTS/CdS/A-TiO_2_/Pt | 6.5 | 11.9 | 0.42 | 2021 | [S18] |
| Mo/CZTS/CdS/TiO_2_-NB/Pt | 6.85 | 14.6 | 0.70 | 2020 | [S6] |
| Mo/CZTS/CdS-MoSx | 6.5 | 14 | 0.60 | 2020 | [S7] |
| Mo/CZTS/CdS/HfO_2_/Pt | Seawater | 12 | 0.75 | 2018 | [S19] |
| Mo/CZTS/Mo/TiO_2_-s/Pt | 6.85 | 13 | 0.63 | 2016 | [S20] |
| Mo/CZTS/CdS/Pt | 6.0 | 3 | 0.58 | 2010 | [S21] |


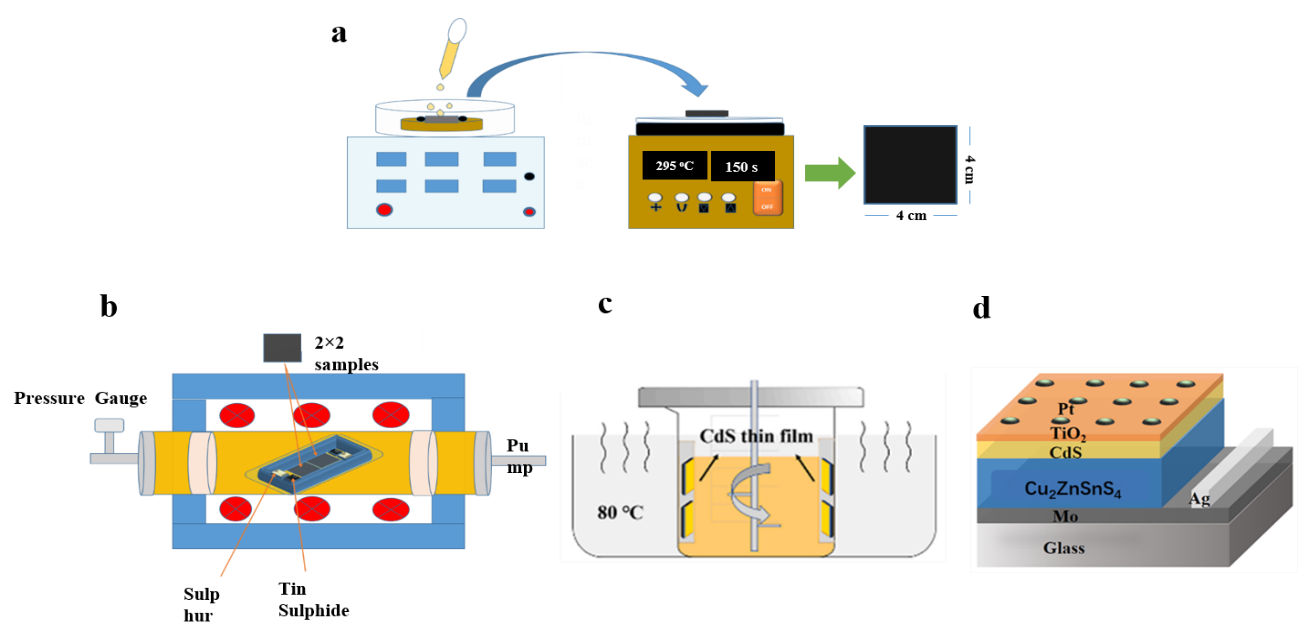


**Fig. S1** Schematic illustration of PSLE based Mo/CZTS/CdS/TiO_2_/Pt photocathode preparation. **a** spin Coating of CZTS Precursor Films **b** Sulfurization of CZTS thin films **c** Chemical bath deposition (CBD) method for CdS buffer layer deposition **d** Final CZTS photocathode after TiO_2_ and Pt depositions


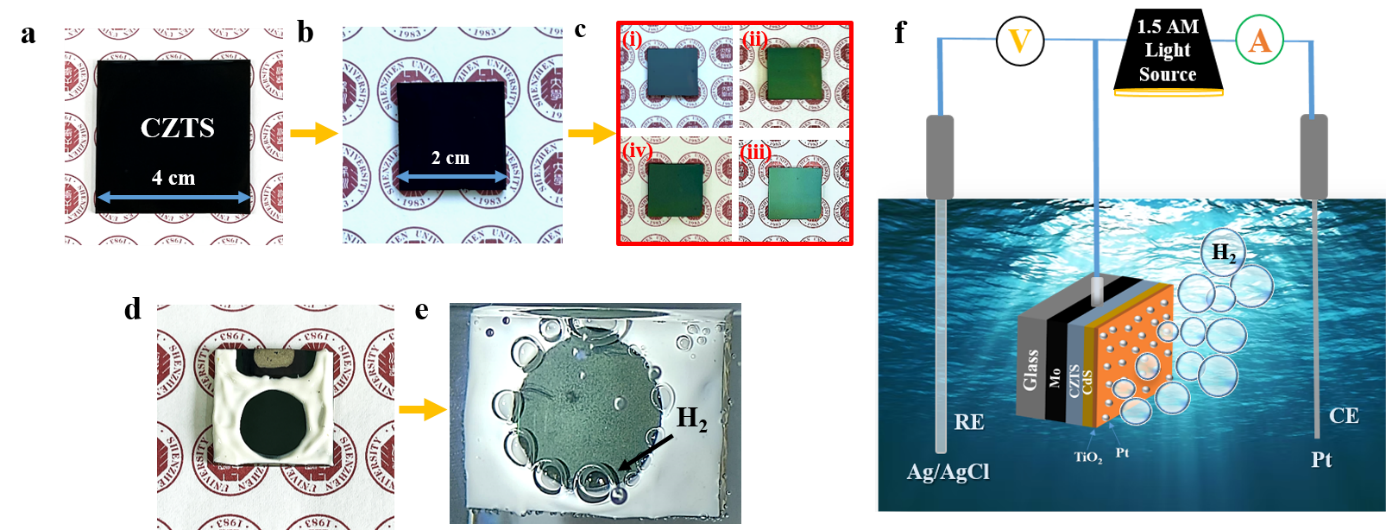


**Fig. S2** Device preparation and testing **a, b** PSLE treated Spin coated 4×4 and 2×2 CZTS precursor films over Mo substrate **c** represents sulfurization, CdS, TiO_2_ and Pt deposition on CZTS precursor film respectively. **d** Preparation of photocathode by adding Ag, **e** Prominent H_2_ bubbles are emerging while testing of as prepared Mo/CZTS/CdS/TiO_2_/Pt Photocathode and **f** Schematic of typical 3-electrode PEC water-splitting configuration

**
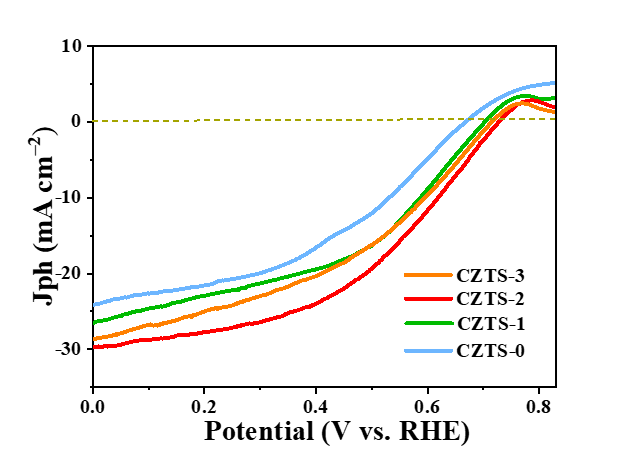
**

**Fig. S3** Dark and continuous light illumination derived *J-V* curves of CZTS-0,1,2 and 3 photocathodes


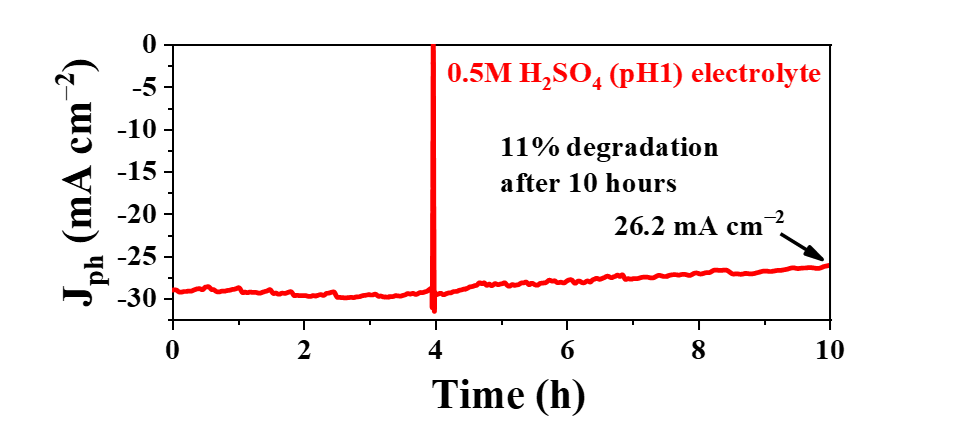


**Fig. S4** Stability test of Mo/CZTS-2/CdS/TiO_2_/Pt photocathode in 0.5M H_2_SO_4_ electrolyte


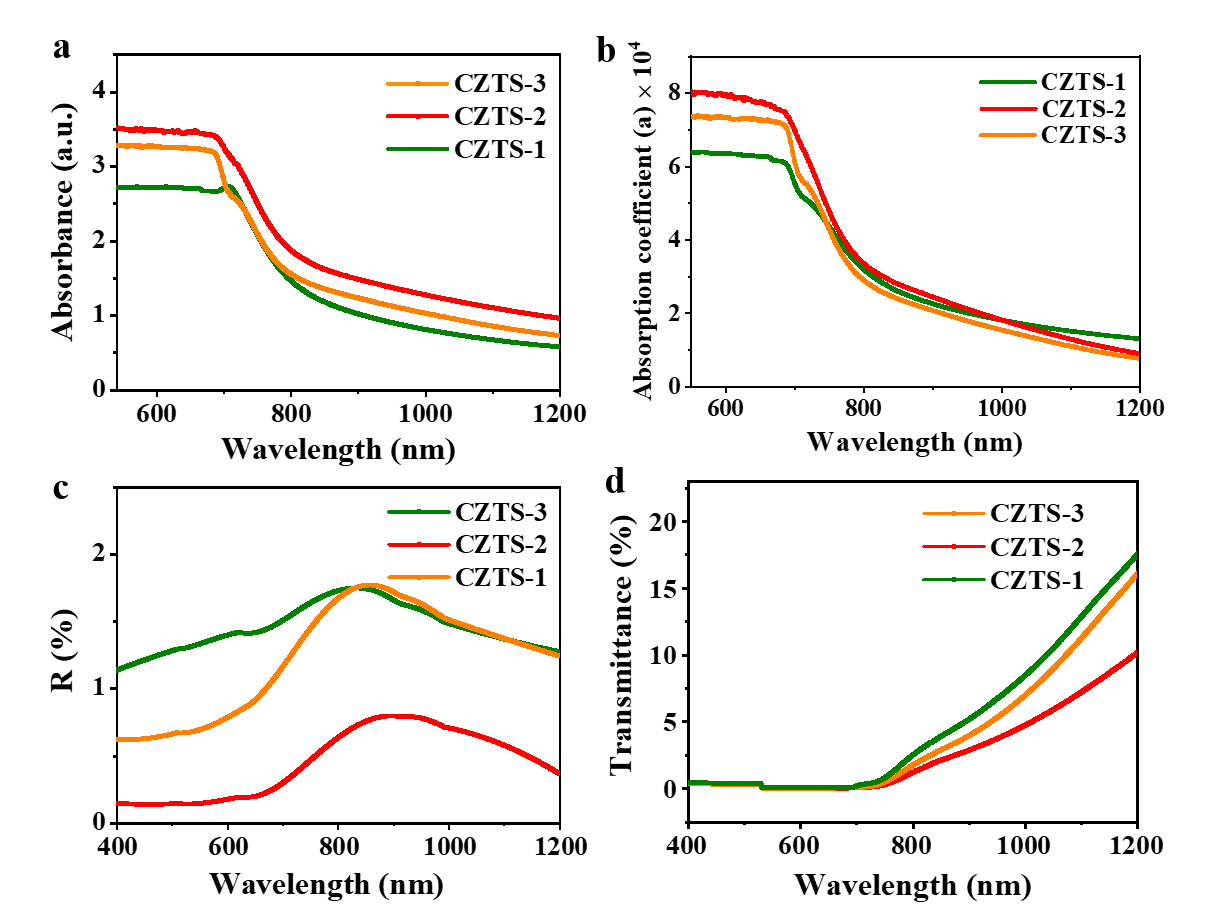


**Fig. S5** Light harvesting related characterizations results of CZTS-1, CZTS-2 and CZTS-3 thin films. **a** Absorbance, **b** absorbance coefficient, **c** reflectance, and **d** transmittance


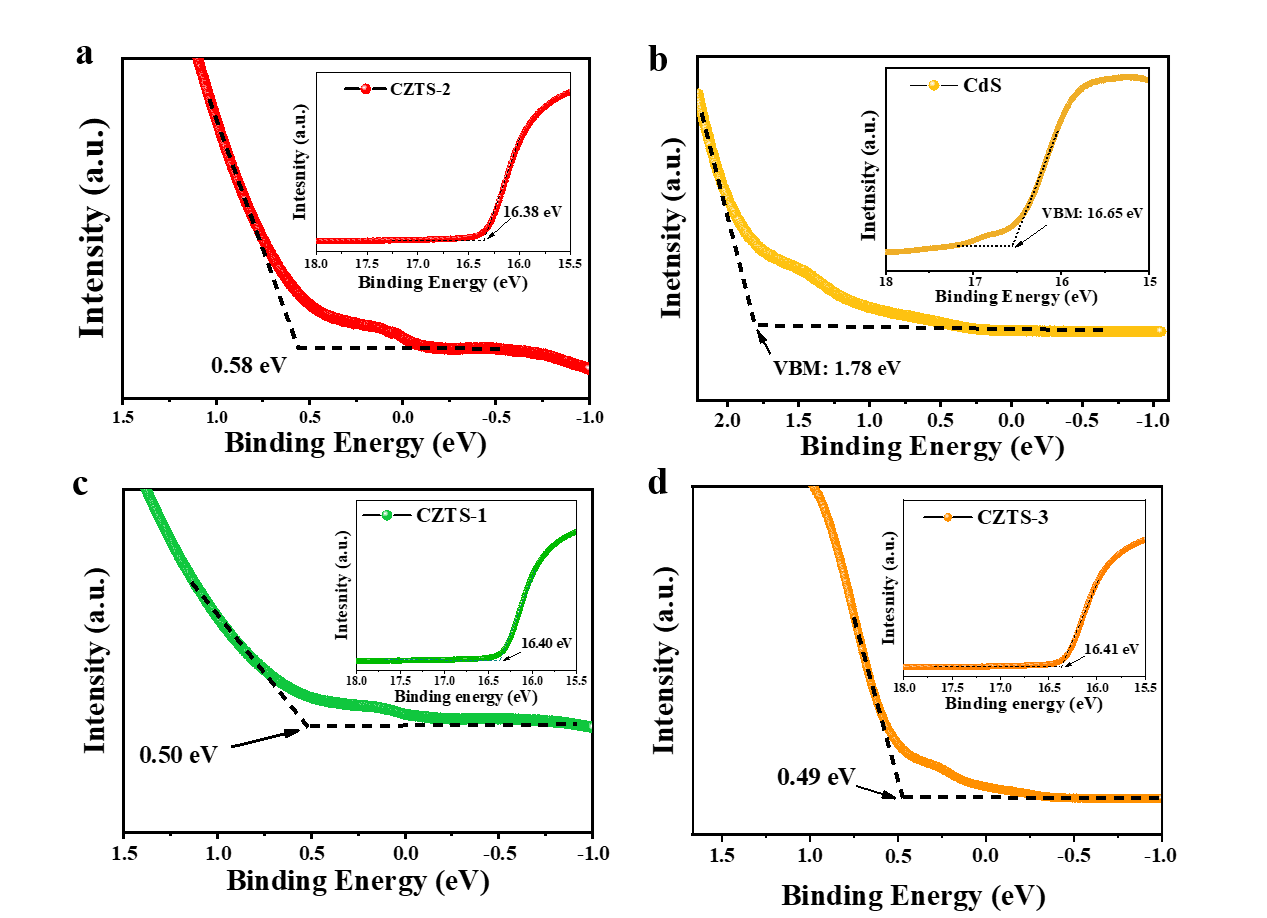


**Fig. S6** UPS characterizations derived V_B_ positions and SEC edges of **a** CZTS-2 **b** CdS **c** CZTS-1 and **d** CZTS-3 thin films


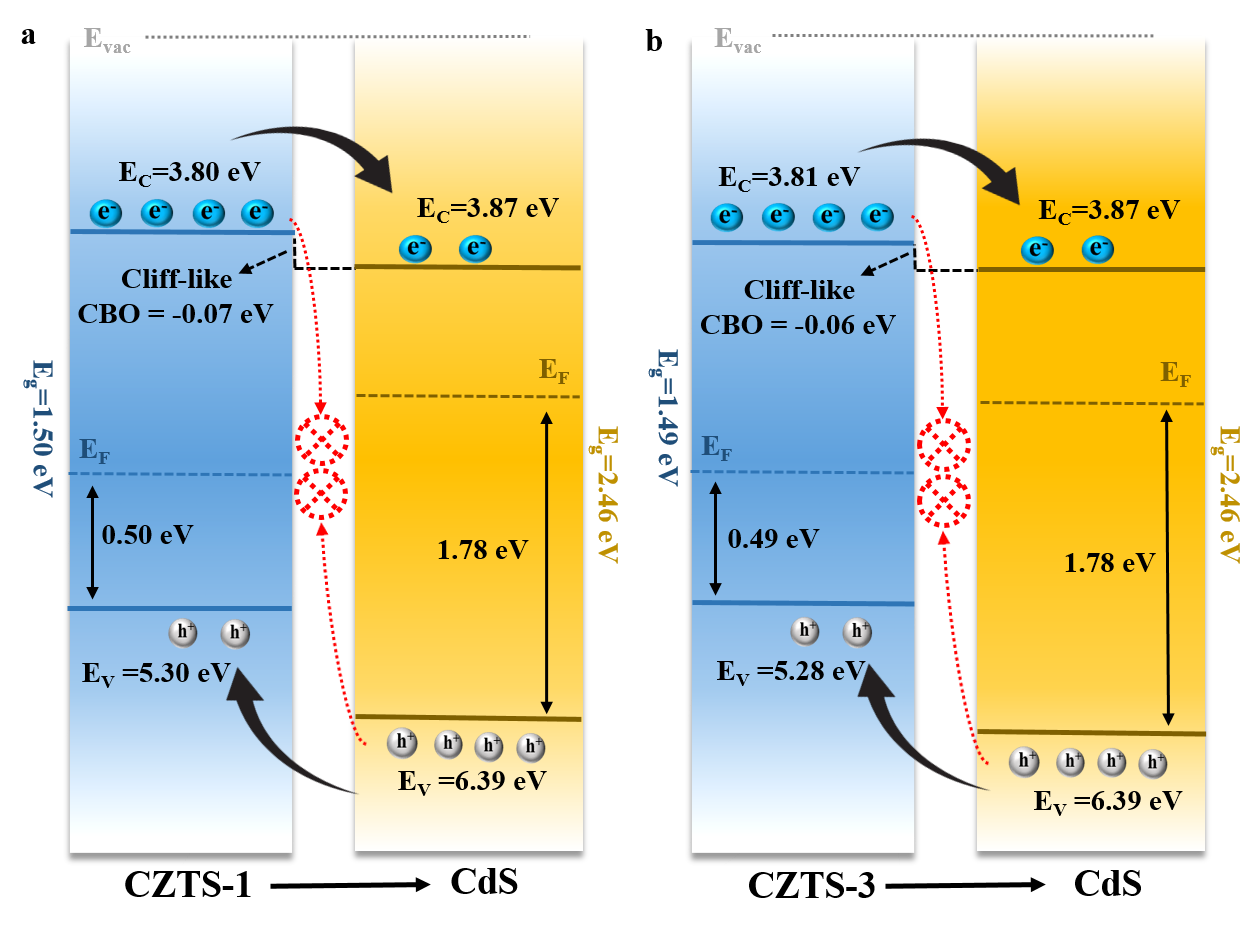


**Fig. S7** Schematic illustration of energy band alignment of **a** CZTS-1/CdS and **b** CZTS-3/CdS hetrostructures


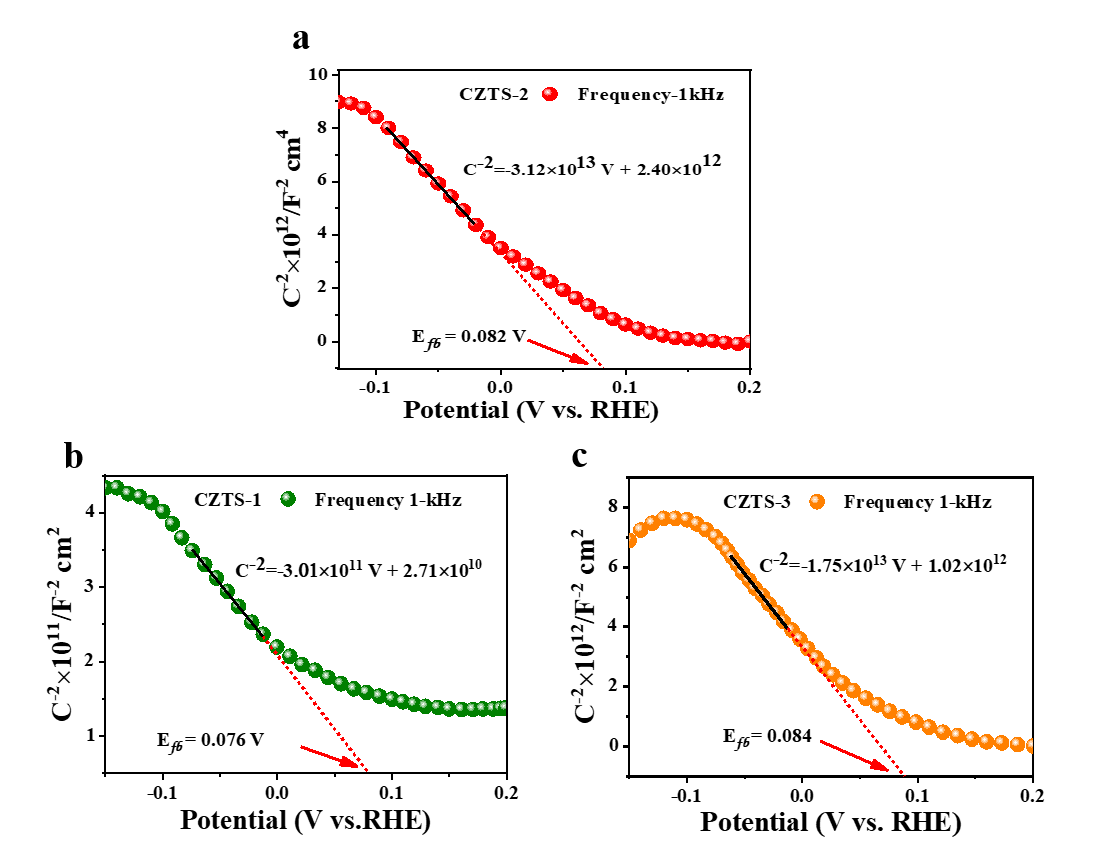


**Fig. S8** *M–S* plots of **a** CZTS-2 and **b** CZTS-1 and **c** CZTS-3 at frequency of 1-kHz


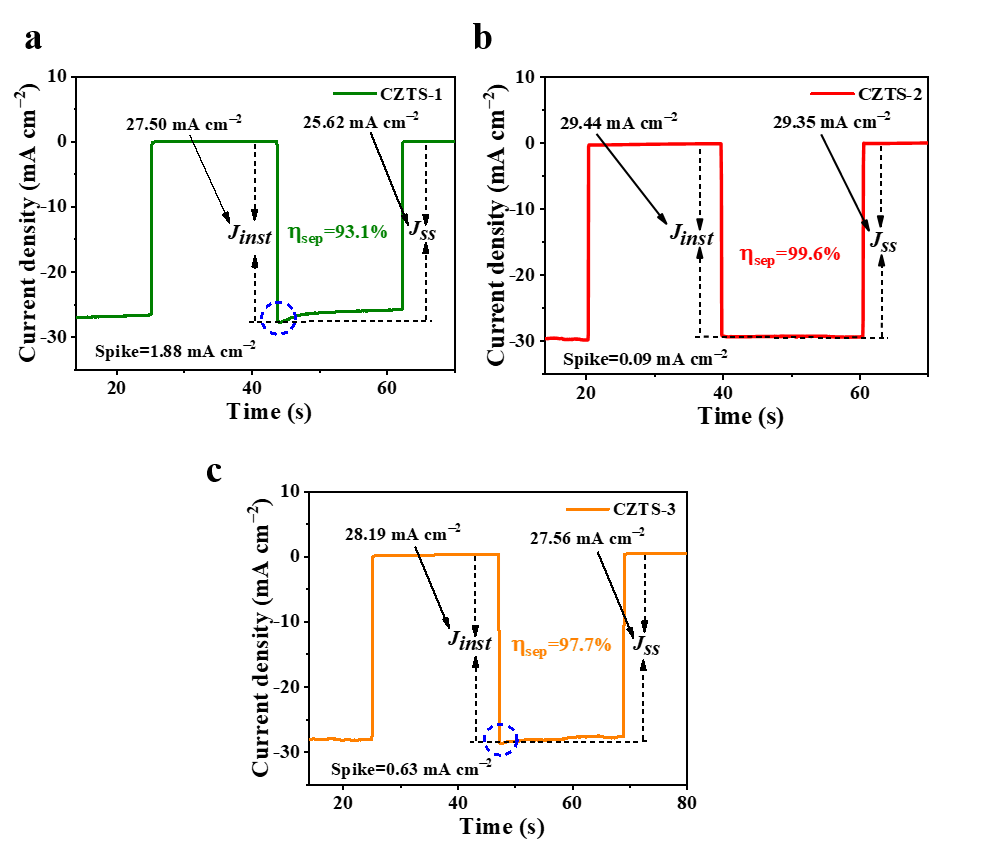


**Fig. S9** Charge separation efficiency calculations using Transient photocurrent response of, **a** CZTS-1, and **b** CZTS-2, and **c** CZTS-3 photocathodes

**
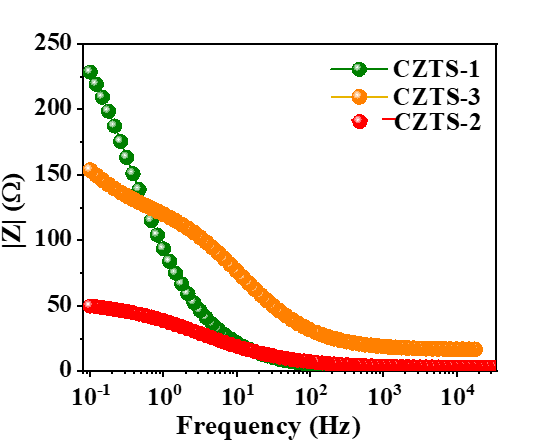
**

**Fig. S10** Nyquist plot of CZTS-1, CZTS-2, and CZTS-3 photocathodes


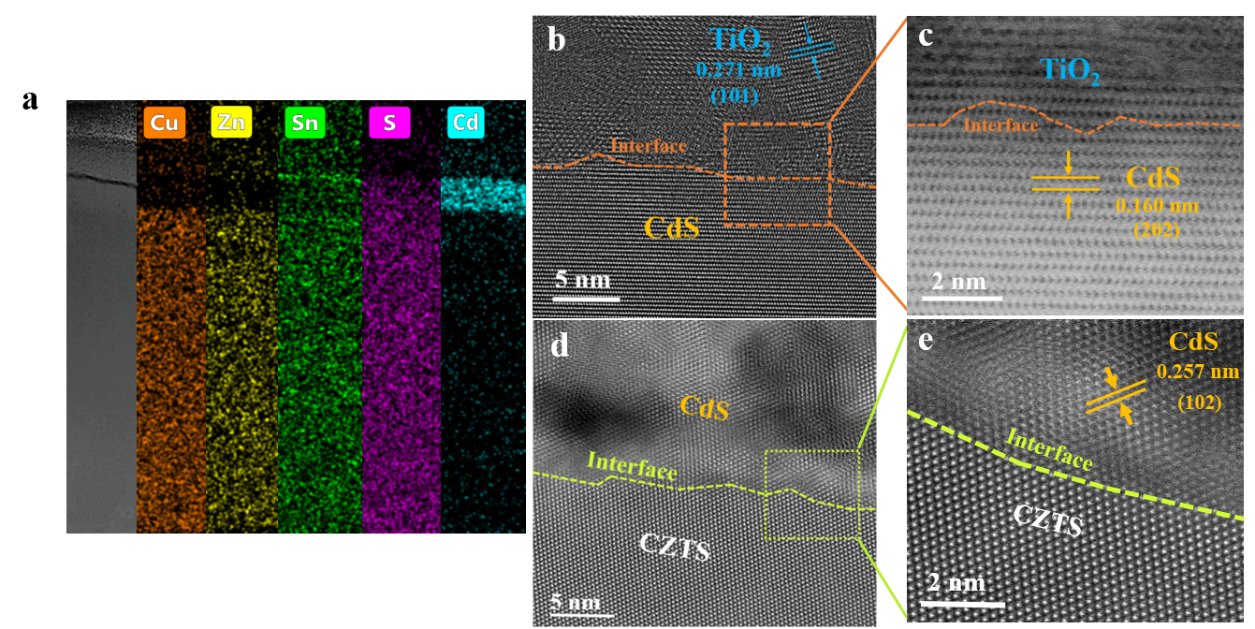


**Fig. S11** Microstructure and elemental mapping information. **a**TEM-coupled EDS elemental mappings of Cu, Zn, Sn, S, Cd **b,c** CdS/TiO_2_ and **d,e** CZTS/CdS interfaces
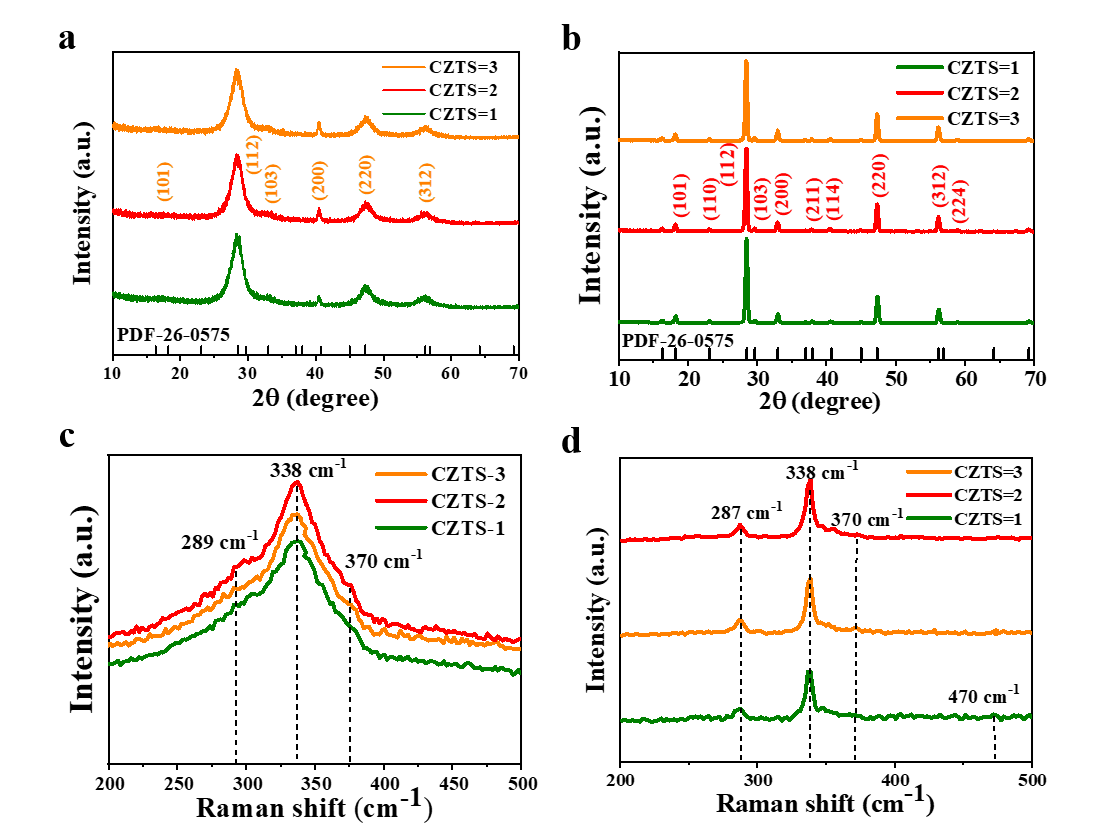


**Fig. S12** Crystal structure information. **a** XRD of CZTS-1, 2 and 3 thin films after spin coating and **b** after sulfurization. **c** Raman spectroscopy of CZTS-1, 2 and 3 thin films after spin coating and **d** after sulfurization


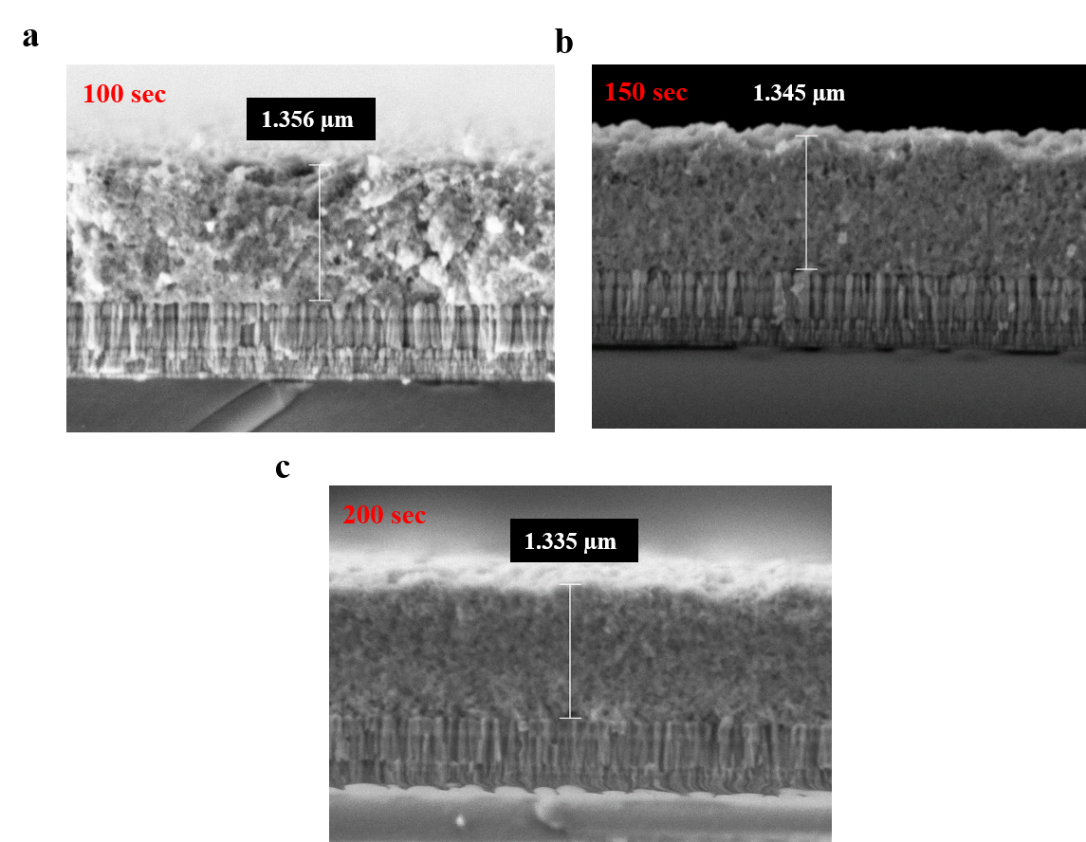


**Fig. S13** The cross sectional SEM images of precursor seed layers at **a** 100 seconds, **b** 150 seconds and **c** 200 seconds annealing duration at 295 ^o^C temperature


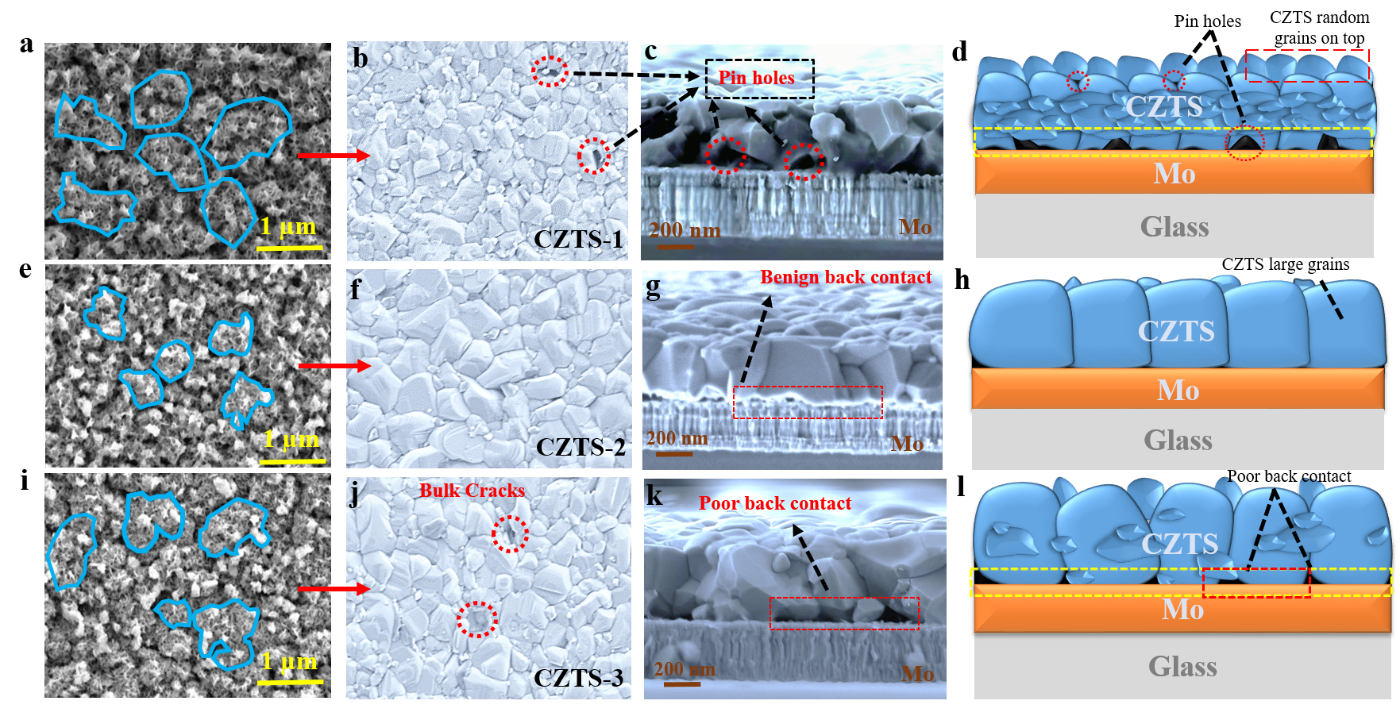


**Fig. S14** The SEM analyses of PSLE strategy based prepared CZTS thin films depict the re-crystallization process from seed layer to sulfurized thin films and corresponding schematic diagrams. **a-d** CZTS-1, **e-h** CZTS-2 and **i-l** CZTS-3


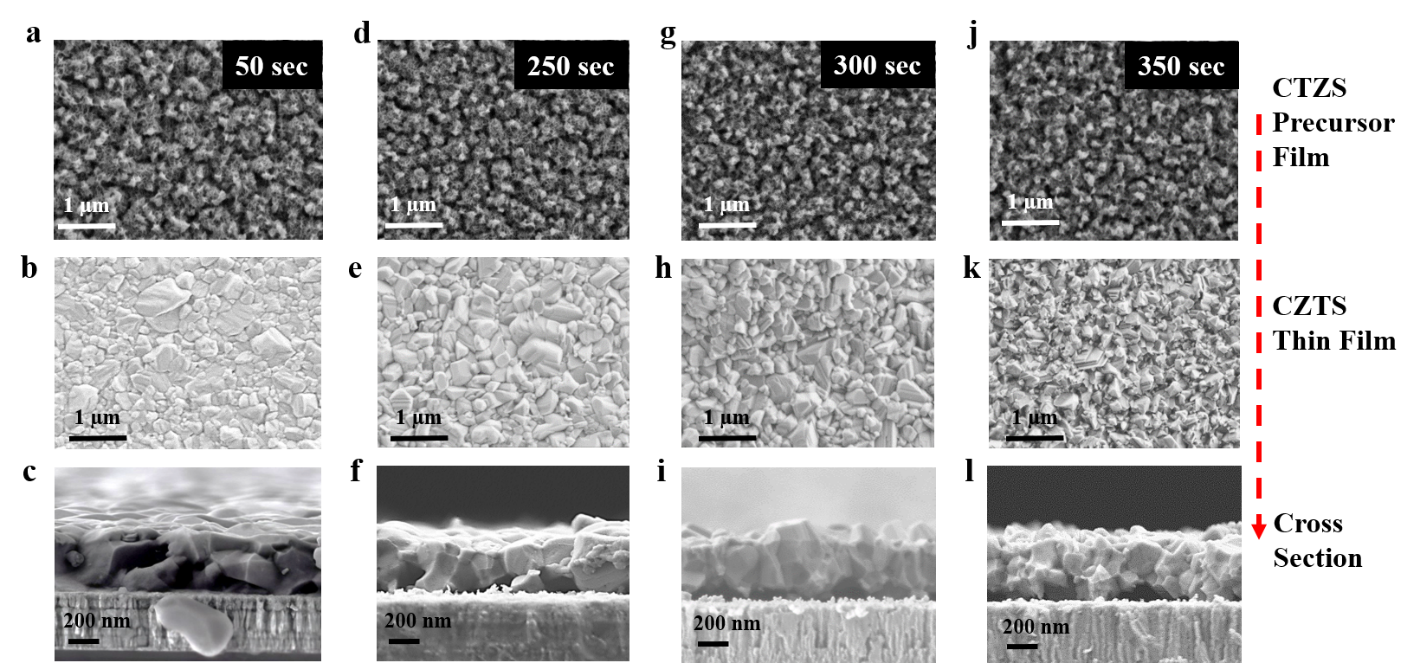


**Fig. S15** SEM analyses of cross section and surface of CZTS thin films after sulfurization at **a-c** 50 seconds, **d-f** 250 seconds, **g-i** 300 seconds, and **j-l** 350 seconds annealing duration


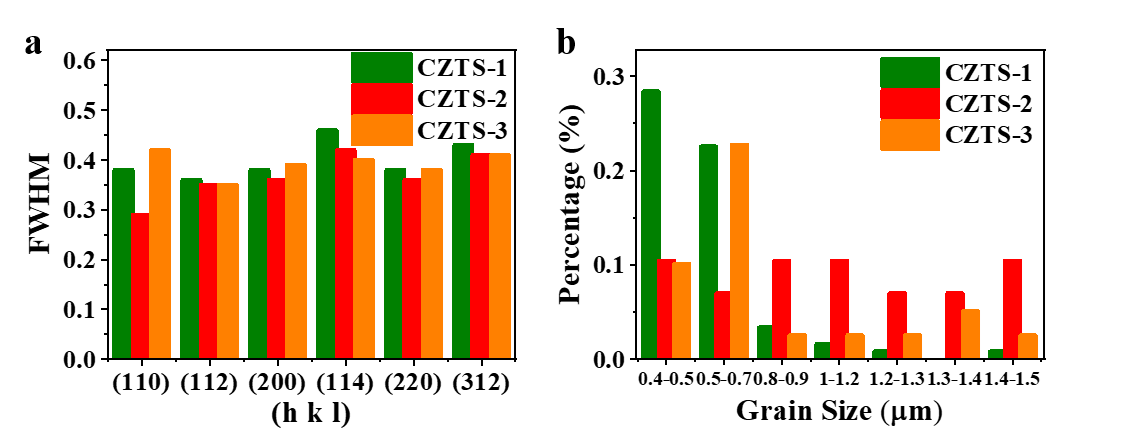


**Fig. S16** Grain size calculation **a** FWHM vs, hkl graph of CZTS-1,2 and 3 thin films and **b** calculated grain sizes of these thin films


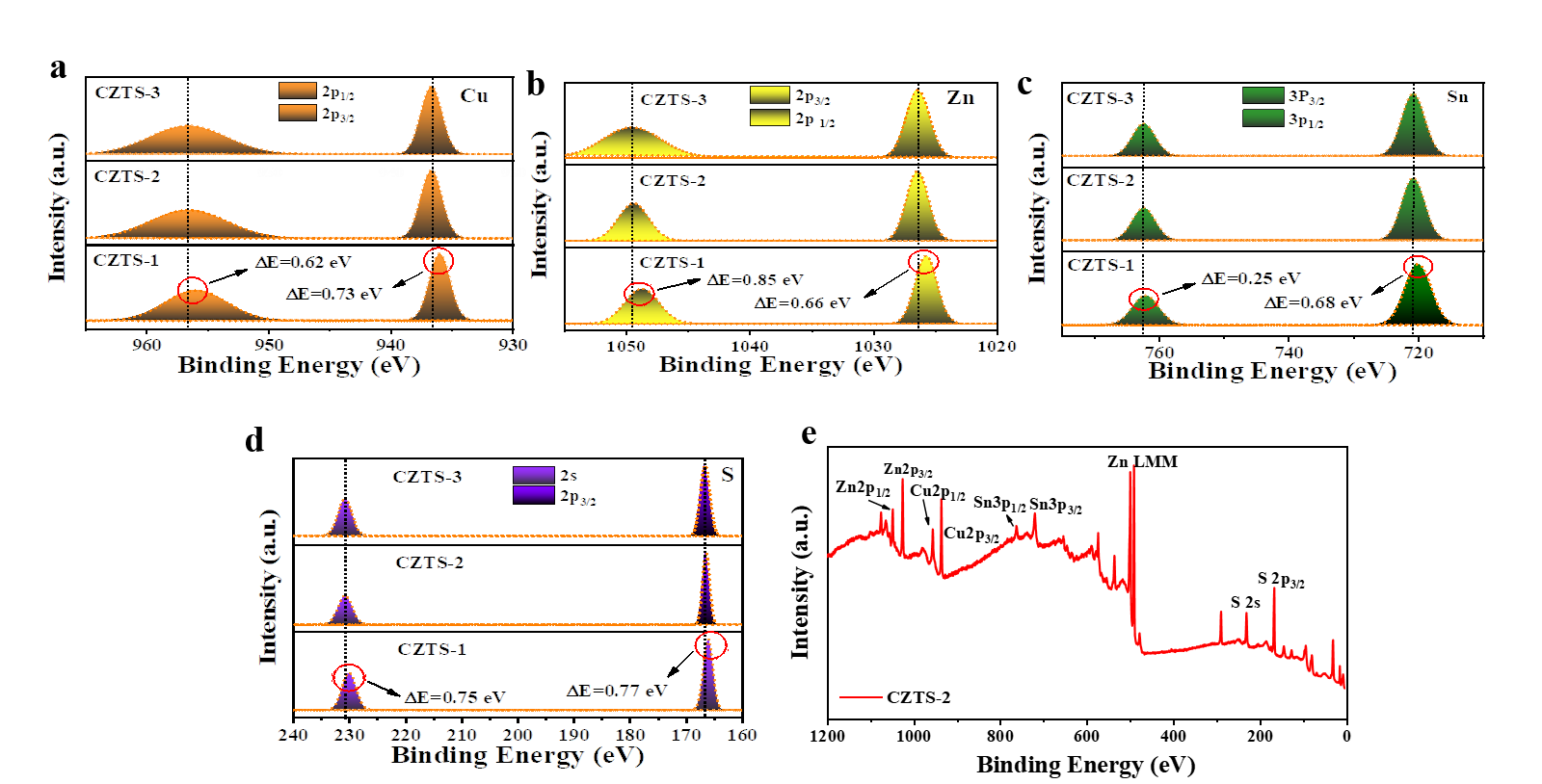


**Fig. S17** XPS characterization. **a-d** comparison of elemental composition of CZTS-1,2 and 3 thin films, **e** Full scan spectra of CZTS-2 thin film


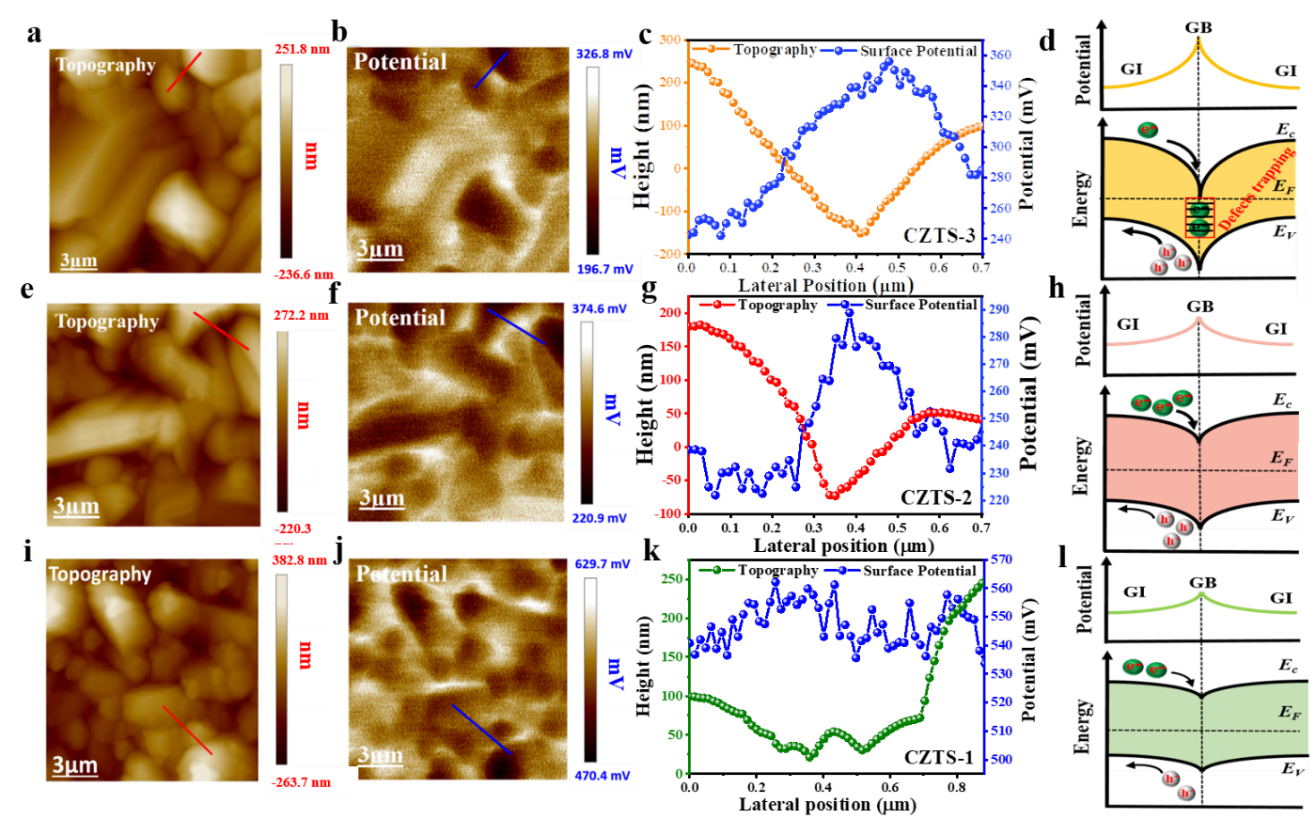


**Fig. S18** KPFM scanning surface topography, potential, CPD maps and corresponding schematic diagrams of the energy band structure of **a-d** CZTS-3, **e-h** CZTS-2 and **i-l** CZTS-1 thin films


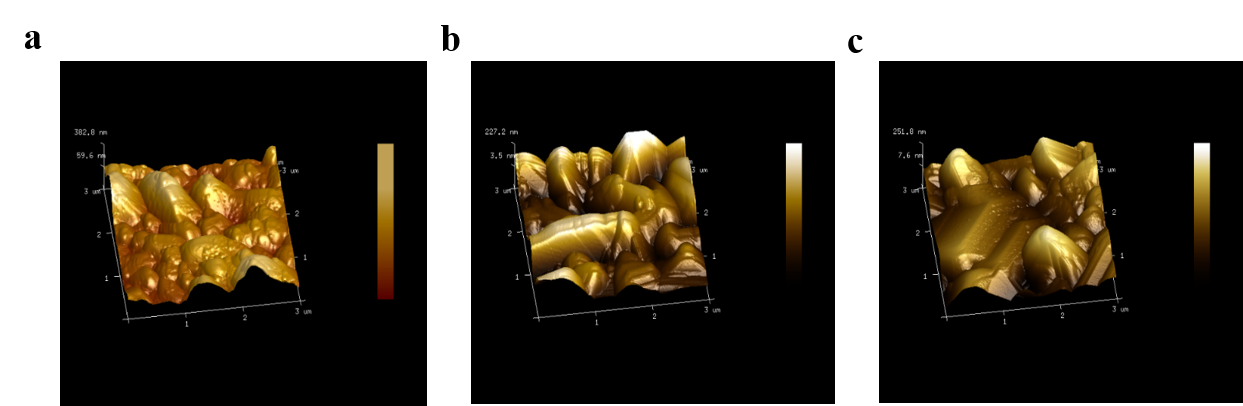


**Fig. S19** 3D potential mapping of **a** CZTS-1, **b** CZTS-2 and **c** CZTS-3


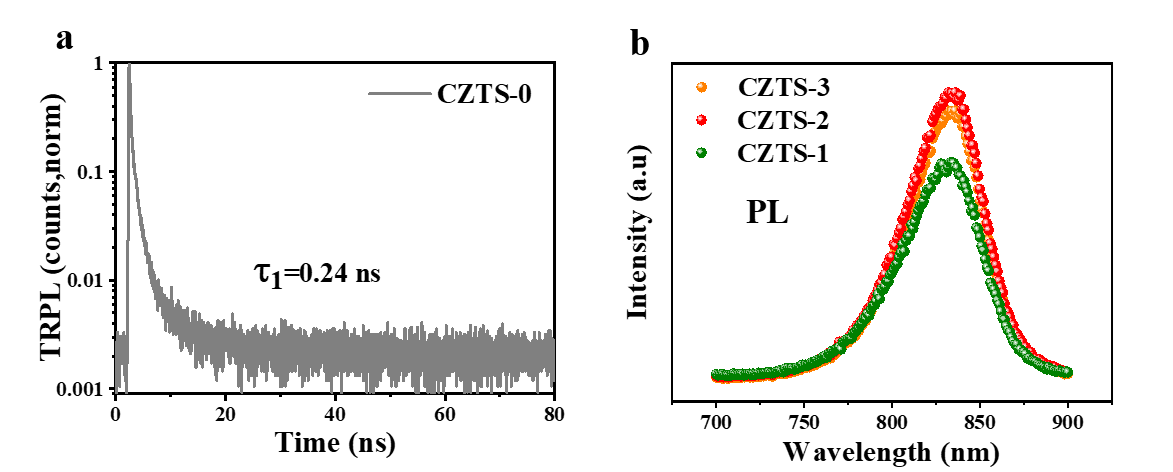


**Fig. S20** **a** TRPL plot of CZTS-0 thin film and **b** PL spectra of CZTS-1,2 and 3 thin films


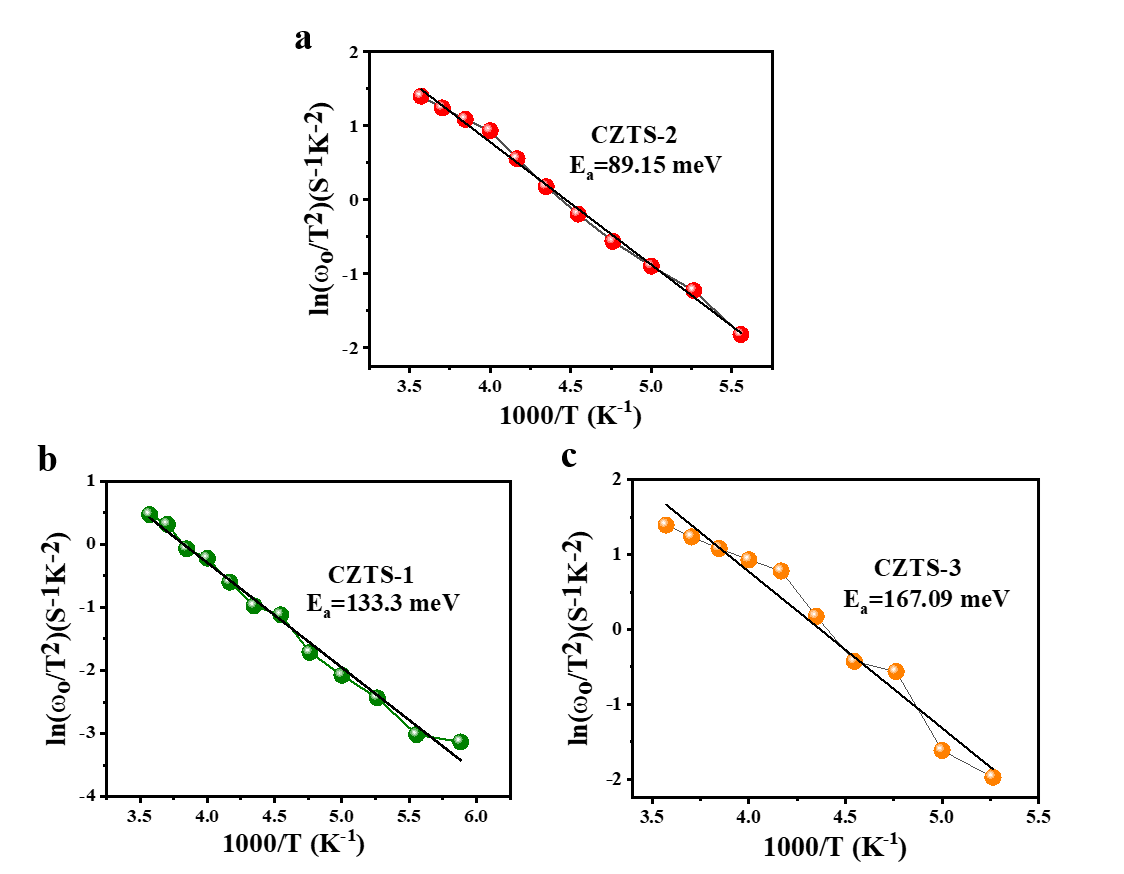


**Fig. S21** Arrhenius plots of the characteristic frequencies to obtain the defect activation energy. **a** PV-CZTS-2, **b** PV-CZTS-1 and **c** PV-CZTS-3 photovoltaic devices


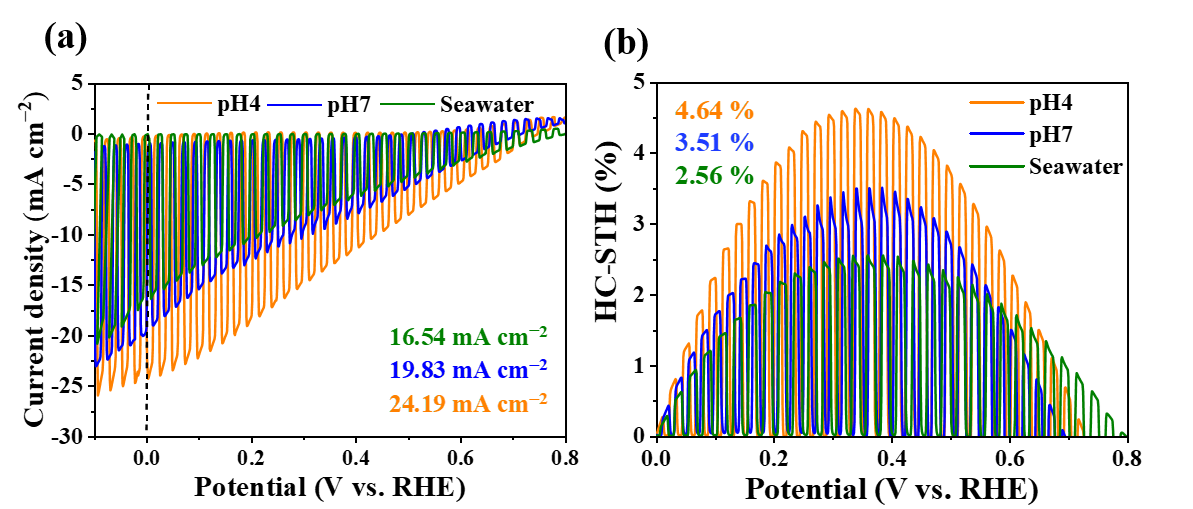


**Fig. S22** PEC performance of Mo/CZTS-2/CdS/TiO_2_/Pt photocathode in pH4, pH7 and seawater electrolytes, **a** photocurrent density and **b** HC-STH efficiency


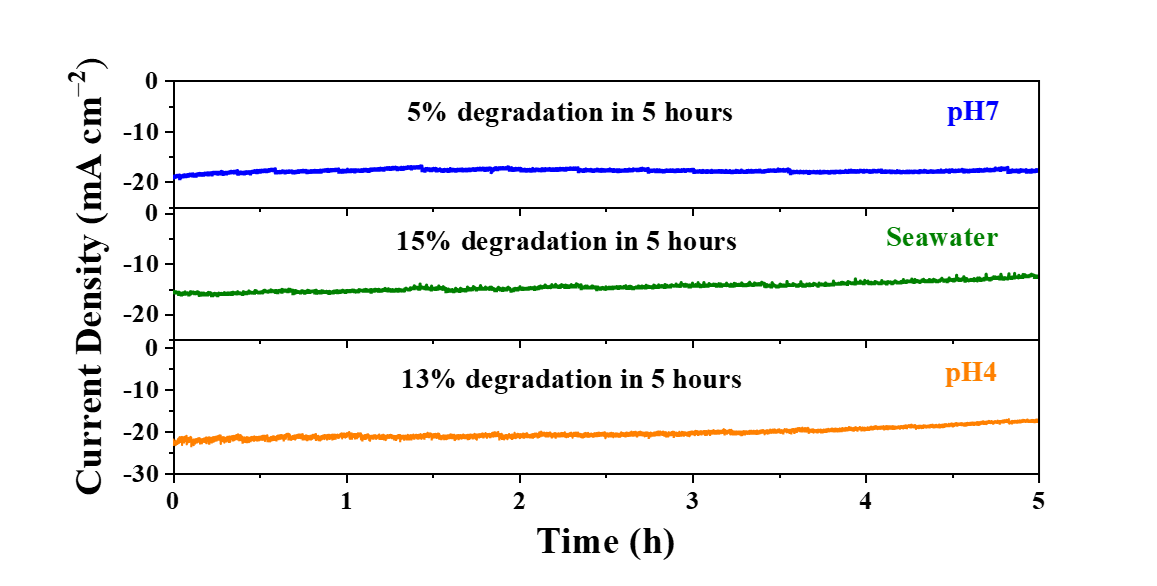


**Fig. S23** Stability test of champion CZTS-2 photocathode in seawater electrolyte under AM 1.5G solar simulated illumination

**Supplementary References**

1. M. Tanaka, Y. Hirose, Y. Harada, M. Takahashi, Y. Sakata et al., Fabrication of Cu_2_ZnSnS_4_ (CZTS) by co-electrodeposition of Cu-Zn-Sn alloys, and effect of chemical composition of CZTS on their photoelectrochemical water splitting. Results Chem. **5**, 100900 (2023). [https://doi.org/10.1016/j.rechem.2023.100900](https://doi.org/https://doi.org/10.1016/j.rechem.2023.100900)
2. Y.F. Tay, M. Zhang, S. Zhang, S. Lie, S.Y. Chiam et al., Charge transfer enhancement at the CZTS photocathode interface using ITO for efficient solar water reduction. J. Mater. Chem. A **11**, 26543-26550 (2023). <https://doi.org/10.1039/D3TA05227C>
3. L. Abeykoon, H. Tan, C. Yan, J. Bandara, Significant role of the initial precursor sulfur concentration in the photoelectrochemical hydrogen production of Cu_2_ZnSnS_4_ photocathode prepared by thermal evaporation. J. Nanophotonics **16**, 016001 (2022). https://doi.org/10.1117/1.JNP.16.016001
4. S. Ikeda, T.H. Nguyen, R. Okamoto, M. Remeika, I. Abdellaoui et al., Effects of incorporation of Ag into a kesterite Cu_2_ZnSnS4 thin film on its photoelectrochemical properties for water reduction. Phys. Chem. Chem. Phys. **24**, 468-476 (2022). <https://doi.org/10.1039/D1CP04075H>
5. D. Huang, K. Wang, L. Li, K. Feng, N. An et al., 3.17% efficient Cu_2_ZnSnS_4_–BiVO_4_ integrated tandem cell for standalone overall solar water splitting. Energy Environ. Sci. **14**, 1480-1489 (2021). <https://doi.org/10.1039/D0EE03892J>
6. L. Li, K. Feng, D. Huang, K. Wang, Y. Li et al., Surface plasmon resonance effect of a Pt-nano-particles-modified TiO_2_ nanoball overlayer enables a significant enhancement in efficiency to 3.5% for a Cu_2_ZnSnS_4_-based thin film photocathode used for solar water splitting. Chem. Engin. J. **396**, 125264 (2020). https://doi.org/10.1016/j.cej.2020.125264
7. K. Feng, D. Huang, L. Li, K. Wang, J. Li et al., MoS_x_-CdS/Cu_2_ZnSnS_4_-based thin film photocathode for solar hydrogen evolution from water. Appl. Catalysis B: Environ. **268**, 118438 (2020). [https://doi.org/10.1016/j.apcatb.2019.118438](https://doi.org/https://doi.org/10.1016/j.apcatb.2019.118438)
8. G. Liang, Z. Li, M. Ishaq, Z. Zheng, Z. Su et al., Charge separation enhancement enables record photocurrent density in Cu_2_ZnSn(S,Se)_4_ photocathodes for efficient solar hydrogen production. Adv. Energy Mater. **13**, 2300215 (2023). [https://doi.org/10.1002/aenm.202300215](https://doi.org/https://doi.org/10.1002/aenm.202300215)
9. S. Chen, T. Liu, M. Chen, M. Ishaq, R. Tang et al., Crystal growth promotion and interface optimization enable highly efficient Sb_2_Se_3_ photocathodes for solar hydrogen evolution. Nano Energy **99**, 107417 (2022). [https://doi.org/10.1016/j.nanoen.2022.107417](https://doi.org/https://doi.org/10.1016/j.nanoen.2022.107417)
10. J. Song, B. Teymur, Y. Zhou, E. Ngaboyamahina, D.B. Mitzi, Porous Cu_2_BaSn(S,Se)_4_ film as a photocathode using non-toxic solvent and a ball-milling approach. ACS Appl. Energy Mater. **4**, 81-87 (2021). <https://doi.org/10.1021/acsaem.0c01892>
11. D. Zhang, M. Du, P. Wang, H. Wang, W. Shi et al., Hole-storage enhanced a-Si photocathodes for efficient hydrogen production. Angew. Chem. Int. Ed. **60**, 11966-11972 (2021). [https://doi.org/10.1002/anie.202100078](https://doi.org/https://doi.org/10.1002/anie.202100078)
12. J. Tan, W. Yang, H. Lee, J. Park, K. Kim et al., Surface restoration of polycrystalline Sb_2_Se_3_ thin films by conjugated molecules enabling high-performance photocathodes for photoelectrochemical water splitting. Appl. Catalysis B: Environ. **286**, 119890 (2021). [https://doi.org/10.1016/j.apcatb.2021.119890](https://doi.org/https://doi.org/10.1016/j.apcatb.2021.119890)
13. J. Zhao, T. Minegishi, H. Kaneko, G. Ma, M. Zhong et al., Efficient hydrogen evolution on (CuInS_2_)_x_(ZnS)_1−x_ solid solution-based photocathodes under simulated sunlight. Chem. Commun. **55**, 470-473 (2019). <https://doi.org/10.1039/C8CC08623K>
14. C.-M. Jiang, S.E. Reyes-Lillo, Y. Liang, Y.-S. Liu, G. Liu et al., Electronic structure and performance bottlenecks of CuFeO_2_ photocathodes. Chem. Mater. **31**, 2524-2534 (2019). <https://doi.org/10.1021/acs.chemmater.9b00009>
15. M. Chen, Y. Liu, C. Li, A. Li, X. Chang et al., Spatial control of cocatalysts and elimination of interfacial defects towards efficient and robust CIGS photocathodes for solar water splitting. Energy Environ. Sci. **11**, 2025-2034 (2018). <https://doi.org/10.1039/C7EE03650G>
16. F. Jiang, Gunawan, T. Harada, Y. Kuang, T. Minegishi et al., Pt/In_2_S_3_/CdS/Cu_2_ZnSnS_4_ thin film as an efficient and stable photocathode for water reduction under sunlight radiation. J. Am. Chem. Soci. **137**, 13691-13697 (2015). <https://doi.org/10.1021/jacs.5b09015>
17. V. Mahalakshmi, D. Venugopal, K. Ramachandran, R. Ramesh, Synthesis of 2D-CZTS nanoplate as photocathode material for efficient PEC water splitting. J. Mater. Sci. Mater. Electron. **33**, 1-11 (2022). <https://doi.org/10.1007/s10854-021-06400-9>
18. L. Li, C. Wang, K. Feng, D. Huang, K. Wang et al., Kesterite Cu_2_ZnSnS_4_ thin-film solar water-splitting photovoltaics for solar seawater desalination. Cell Rep. Phys. Sci. **2**, 100468 (2021). [https://doi.org/10.1016/j.xcrp.2021.100468](https://doi.org/https://doi.org/10.1016/j.xcrp.2021.100468)
19. L. Wu, Cu-based mutlinary sulfide nanomaterials for photocatalytic applications. AIMS Mater. Sci. **10**, 909-933 (2023). <https://doi.org/10.3934/matersci.2023049>
20. W. Yang, Y. Oh, J. Kim, M.J. Jeong, J.H. Park et al., Molecular chemistry-controlled hybrid ink-derived efficient Cu_2_ZnSnS_4_ photocathodes for photoelectrochemical water splitting. ACS Energy Lett. **1**, 1127-1136 (2016). <https://doi.org/10.1021/acsenergylett.6b00453>
21. D. Kang, J.C. Hill, Y. Park, K.-S. Choi, Photoelectrochemical properties and photostabilities of high surface area CuBi_2_O_4_ and Ag-doped CuBi_2_O_4_ photocathodes. Chem. Mater. **28**, 4331-4340 (2016). <https://doi.org/10.1021/acs.chemmater.6b01294>
